# Facile synthesis of low toxicity iron oxide/TiO_2_ nanocomposites with hyperthermic and photo-oxidation properties

**DOI:** 10.1038/s41598-022-11003-3

**Published:** 2022-04-27

**Authors:** Traian Popescu, Christien Oktaviani Matei, Daniela Cristina Culita, Valentin-Adrian Maraloiu, Arpad Mihai Rostas, Lucian Diamandescu, Nicusor Iacob, Tudor Savopol, Monica Cristiana Ilas, Marcel Feder, Andreea-Roxana Lupu, Alexandra Corina Iacoban, Ioana Dorina Vlaicu, Mihaela Georgeta Moisescu

**Affiliations:** 1 National Institute of Materials Physics, Str. Atomistilor 405A, POB MG 7, 077125 Magurele, Ilfov Romania; 2 Biophysics and Cellular Biotechnology Department, Excellence Centre for Research in Biophysics and Cellular Biotechnology, Carol Davila University of Medicine and Pharmacy, 8 Eroii Sanitari Blvd., 050474 Bucharest, Romania; 3 Ilie Murgulescu Institute of Physical Chemistry, Romanian Academy, 202 Splaiul Independentei, 060021 Bucharest, Romania; 4 “Victor Babes” National Institute of Pathology, Splaiul Independentei 99-101, Bucharest, Romania

**Keywords:** Medical research, Materials science

## Abstract

The present study aimed to assess the feasibility of developing low-cost multipurpose iron oxide/TiO_2_ nanocomposites (NCs) for use in combined antitumor therapies and water treatment applications. Larger size (≈ 100 nm) iron oxide nanoparticles (IONPs) formed magnetic core-TiO_2_ shell structures at high Fe/Ti ratios and solid dispersions of IONPs embedded in TiO_2_ matrices when the Fe/Ti ratio was low. When the size of the iron phase was comparable to the size of the crystallized TiO_2_ nanoparticles (≈ 10 nm), the obtained nanocomposites consisted of randomly mixed aggregates of TiO_2_ and IONPs. The best inductive heating and ROS photogeneration properties were shown by the NCs synthesized at 400 °C which contained the minimum amount of α-Fe_2_O_3_ and sufficiently crystallized anatase TiO_2_. Their cytocompatibility was assessed on cultured human and murine fibroblast cells and analyzed in relation to the adsorption of bovine serum albumin from the culture medium onto their surface. The tested nanocomposites showed excellent cytocompatibility to human fibroblast cells. The results also indicated that the environment (i.e. phosphate buffer or culture medium) used to disperse the nanomaterials prior to performing the viability tests can have a significant impact on their cytotoxicity.

## Introduction

The feasibility of using inorganic nanostructures with engineered physicochemical, optoelectronic, and magnetic properties for various biomedical applications (drug carriers, contrast media, sensitizers, active antitumor agents, or adjuvants in cancer therapeutics) as well as for the treatment of wastewaters, has been widely studied and the results are promising^1,2^.

Magnetic hyperthermia (MHT) and semiconductor photodynamic therapy (SCPDT) represent two cancer therapeutic approaches in which nanoparticles (NPs) with magnetic and semiconductor properties exert direct antitumor activity by the generation of heat and reactive oxygen species (ROS) into the tumor tissue, respectively.

In the case of MHT, magnetic nanoparticles heat up when they are exposed to external alternating (AC) magnetic fields^3^. The dissipated heat acts preferentially on tumor cells by destabilizing cell membranes and cytoskeletal organization, promoting protein denaturation, diminishing the repairing of radiation-induced DNA damage, and increasing tumor immunogenicity through heat shock protein and T cell activation^1,4^.

The heating efficiency of the ferrofluids used in MHT applications is characterized by the specific absorption rate (SAR) (heating power generated per unit mass of ferrofluid), defined as^5^:

$$ SAR = c\frac{\Delta T}{{\Delta t}} $$

where *c* is the specific heat capacity of the ferrofluid, measured in JK^−1^ kg^−1^ and Δ*T* is the temperature rise during the time interval Δ*t*. The SAR is experimentally determined from the heating curves, *T(t)*, of magnetic nanoparticle suspensions exposed to alternating magnetic fields. In-depth discussions regarding various methods for SAR calculation can be found in the published literature^5–7^.

One convenient practical way to characterize the heating efficiency of nanoparticles in magnetic hyperthermia is to determine the SAR corresponding to the mass of iron oxide nanoparticles (IONPs) in the sample, according to^8^:

$${SAR}_{IONPs}=\frac{{SAR}_{s}{m}_{s}}{{m}_{IONPs}}={c}_{s}\frac{{m}_{s}\Delta T}{{m}_{IONPs}\Delta t}$$

where ${m}_{s}$ and ${c}_{s}$ are the mass and the specific heat of the ferrofluid sample.

The specific heat, ${c}_{s}$, of the magnetic fluid can be approximated by^9^:

$${c}_{s}=\sum_{j=1}^{j=n}{m}_{j}{c}_{j}$$

where *m*_*j*_ and *c*_*j*_ represent the mass and the specific heat capacity for each constituent of ferrofluid.

Besides good AC heating properties, magnetic nanomaterials for hyperthermia should also have good biocompatibility. Iron oxide nanoparticles like Fe_3_O_4_ and γ-Fe_2_O_3_ proved to be promising MHT agents^10–13^.

In the case of SCPDT, photoexcited semiconductor nanomaterials called photosensitizers exert antitumor action through generated oxygen radicals. Due to the high complexity of the cell-nanomaterial interaction, the action sites and consequently the cellular (morphological and biochemical) and molecular (on genes, proteins, and enzymes) effects of SCPDT-induced oxidative stress are still poorly understood^14,15^.

Titanium dioxide (TiO_2_) nanoparticles can act as photosensitizers due to their photocatalytic properties. The photoexcitation of nanosized TiO_2_ with UV radiation (λ < 385 nm) leads to the generation of negatively charged electrons (e^−^) and positively charged holes (h^+^) at its surface. Under appropriate conditions, the photogenerated charge carriers undergo interfacial transfer and engage in redox reactions with adsorbed molecular oxygen, water molecules, and hydroxide ions, producing various types of ROS such as superoxide, hydrogen peroxide, and hydroxyl radicals. The pathways of ROS formation at the surface of photoexcited TiO_2_ catalysts in aerated aqueous environments have been discussed in previous literature on semiconductor photocatalysis^16–18^.

The first reports on the phototoxic effect of TiO_2_ nanoparticles on tumor cells were published by Cai et al. in the early nineties^19–21^. The studies that followed concerned bare TiO_2_ nanoparticles as well as TiO_2_/photosensitizer-molecule nano-conjugates and provided supporting evidence for the use of TiO_2_ nanostructures in SCPDT^22–24^. In the absence of activating light, TiO_2_ exhibits low toxicity and is considered for use in a variety of biomedical applications^25–28^.

As regards the combined effects of conventional hyperthermia and photodynamic therapy (PDT), the few published reports on the subject revealed that the occurrence of a synergic action depended on the sequence of application of the two antitumor factors and encouraged the research on the subject^29^.

The goal of the present study was to primarily assess the feasibility of developing low-cost iron oxide/TiO_2_ nanocomposites (NCs) with reduced toxicity and adjustable efficiency of heat generation under alternating magnetic fields and ROS production under UV irradiation. Depending on their phase content and microscopic phase distribution, such nanocomposites are promising for use in combined MHT and SCPDT as well as for wastewater treatment applications based on photocatalytic decomposition of pollutants and subsequent magnetic removal of the photocatalyst.

Most of the few previous studies on iron oxide (Fe_3_O_4_, α-Fe_2_O_3_)–TiO_2_ composites concern the photocatalytic activity of core–shell nanostructures evaluated by decomposition of model pollutants^30–39^. Some authors proposed the use of such heterostructures as supercapacitors^35^, affinity probes for the analysis of phosphopeptides^40,41^, and bacteria photo-killing agents^42^. Very few reports describe in-depth analyses of the phase composition and structural, magnetic, and optical properties of the obtained iron oxide-TiO_2_ nanocomposites with respect to the used synthesis methods and conditions^43^.

For the purpose of the present study, three types of iron oxide nanoparticles were used to synthesize nanocomposites with different Fe/Ti ratios. A combination of multiple structural characterization methods and elemental mapping was employed to accurately identify the iron and titanium phases and their distribution in the analyzed composites with respect to thermal treatment conditions and relative particle size. The impact of these parameters on the functional characteristics of the obtained nanocomposites, required for their use in MHT and PDT, was assessed by analyzing the kinetics of the AC magnetic heating and ROS photogeneration under UV irradiation. In what regards the biocompatibility of the studied NCs in the absence of the activation factors (AC magnetic fields or UV radiation), it is known that the in vitro and in vivo biological effects of inorganic nanomaterials are mediated by the biomolecules spontaneously adsorbed on their surface, especially proteins that form the “protein corona” (PC), which define the “biological identity” of the nanomaterial^44,45^. Previous studies have shown that PC can either reduce or increase the toxicity of nanomaterials^46–48^. In this context, the adsorption of serum albumin onto the synthesized NCs was studied with respect to their Fe/Ti ratio and surface chemistry. The in vitro cytotoxicity of the protein-coated NCs was evaluated on human and murine fibroblast cells.

The present study tackles perspectives on synthesizing multifunctional iron oxide/TiO_2_ nanocomposites with adjustable magnetic and optical properties, suitable for facile bio-compatibilization or functionalization with organic photosensitizers, therapeutic molecules or pollutant-binding agents.

## Results

**Figure 1:**
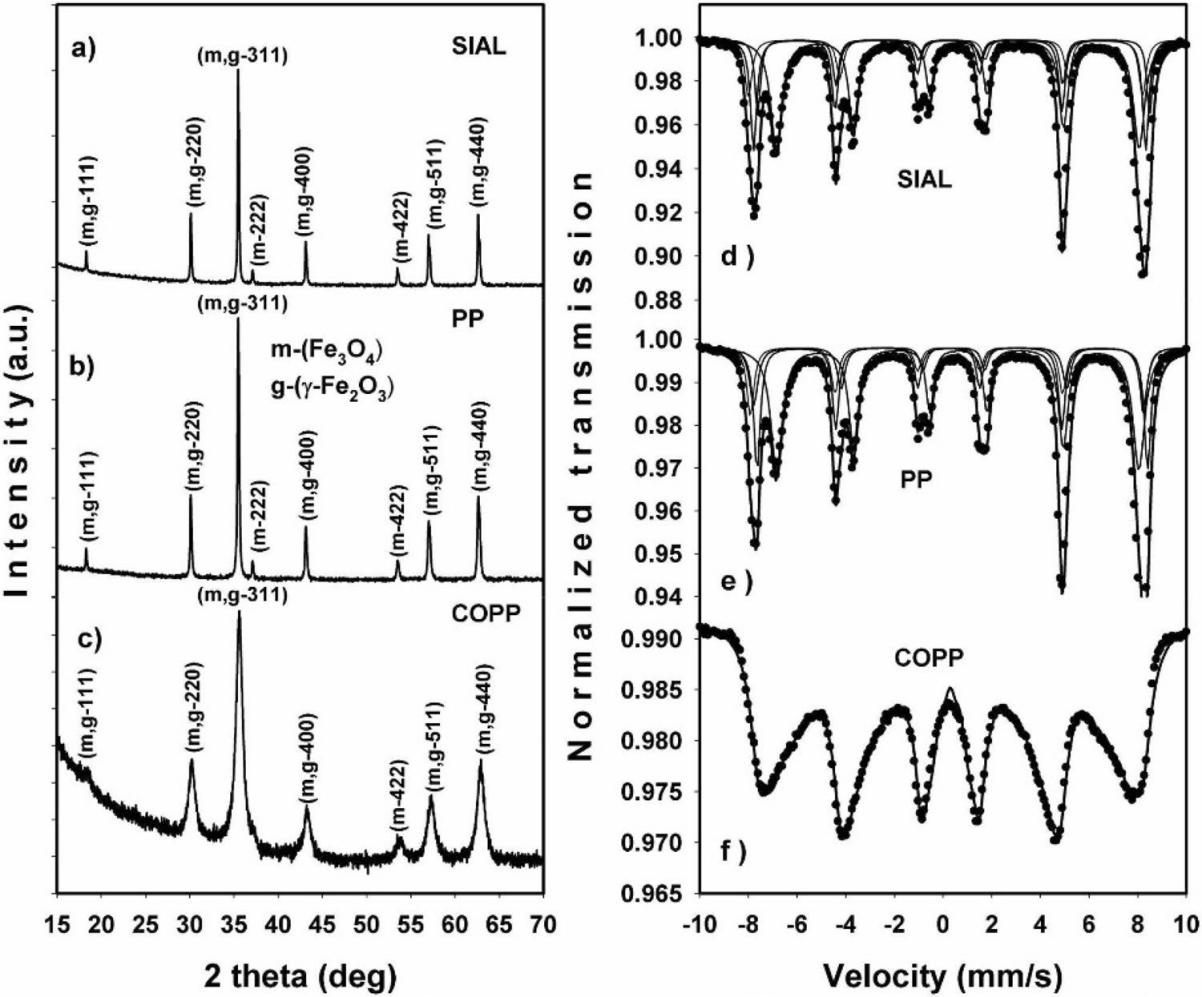
X-ray diffractograms (**a**–**c**) and fitted Mössbauer spectra (**d**–**f**) of the initial iron oxides.

The X-ray diffraction (XRD) and Mössbauer spectroscopy analyses revealed the structure, phase composition and crystallite size of the SIAL, PP and COPP iron oxides. The Rietveld refinement of the XRD data (Fig. 1a–c) indicated mixed phase compositions for all samples: SIAL (76% Fe_3_O_4_, 24% γ-Fe_2_O_3_), PP (77% Fe_3_O_4_, 23% γ-Fe_2_O_3_) and COPP (71% Fe_3_O_4_, 29% γ-Fe_2_O_3_) with crystallite sizes of 109 nm, 90 nm and 9 nm, respectively. The reported sizes represent averages over the mean sizes corresponding to each phase present in the studied iron oxide samples.

These results were well confirmed by Mössbauer spectroscopy determinations. The fitted Mössbauer spectra of the SIAL, PP and COPP samples are shown in Fig. 1d–f.

The main hyperfine Mössbauer parameters, isomer shift (IS), quadrupole splitting (∆E_Q_) and hyperfine magnetic field (H_hf_) are displayed in Table 1, as well as the site assignment, sublattice areas and relative abundance of phases computed in the hypothesis of Lorentzian line shape. Two magnetic phases with iron atoms localized in tetrahedral positions (A) and octahedral positions (B), magnetite (Fe_3_O_4_) and respectively maghemite (γ-Fe_2_O_3_), were identified^49^ from the Mössbauer spectra of both SIAL and PP IONPs. Their relative amounts, revealed by the Lorentzian fit of Mössbauer data, is in good agreement with the XRD results. The Mössbauer spectrum of the COPP sample (Fig. 1f) is collapsed due to the small particle size effect^50^. In this case, the best fit was obtained with a magnetic hyperfine distribution, no phase separation being possible in a computer run.

**Table 1:** Mössbauer fit results for the SIAL, PP and COPP initial iron oxides.

Sample	IS* (mm/s)	∆E_Q_ (mm/s)	H_hf_ (T)	Site assignment/area (%)	Relative abundance (%)
SIAL	0.387	0.186	49.7	Magnetite (A)/25	70
	0.573	− 0.032	46.1	Magnetite (B)/45	
	0.330	− 0.017	48.4	Maghemite (A)/12	30
	0.346	− 0.362	50.0	Maghemite (B)/18	
PP	0.358	0.126	49.7	Magnetite (A)/31	72
	0.587	− 0.018	46.0	Magnetite (B)/41	
	0.492	− 0.217	48.2	Maghemite (A)/11	28
	0.318	− 0.198	49.8	Maghemite (B)/17	
COPP	0.384	0.013	8.6–48.5	Hyperfine fields distribution	100
Errors	± 0.002	± 0.004	± 0.03		± 1.0

**Figure 2:**
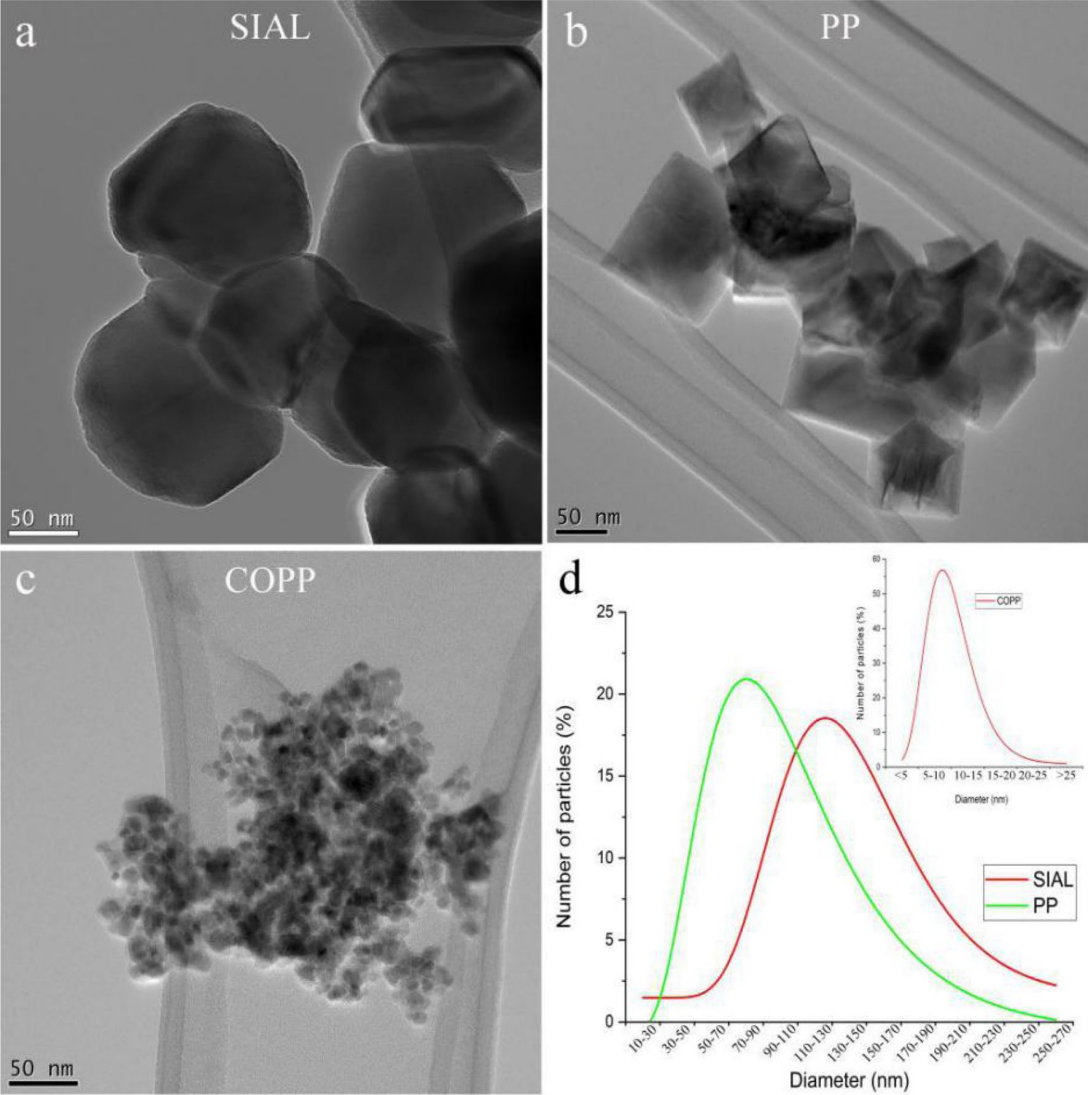
CTEM images of the initial iron oxide nanoparticles (**a**–**c**) and their size distributions fitted with a log–normal function (**d**).

The morphology and size of the initial iron oxide nanoparticles (IONPs) were investigated by CTEM. The observed particle morphology was different for the three samples (Fig. 2a–c). While SIAL and COPP consisted of irregularly-shaped quasi-spherical nanoparticles, the PP sample showed faceted NPs with cubical or octahedral shapes. The acquired images revealed monomodal particle-size distributions (SDs) ranging between 30 and 270 nm for SIAL, 10–230 nm for PP and 3–27 nm for COPP, the corresponding mean diameters being 130 nm (SIAL), 97 nm (PP) and 10 nm (COPP), as shown in Fig. 2d. It is noticeable that the size-distribution of the COPP sample is narrow in comparison to the SDs of the SIAL and PP nanomaterials. The observed mean sizes were in good agreement with the values obtained by Rietveld refinement of the XRD data.

**Figure 3:**
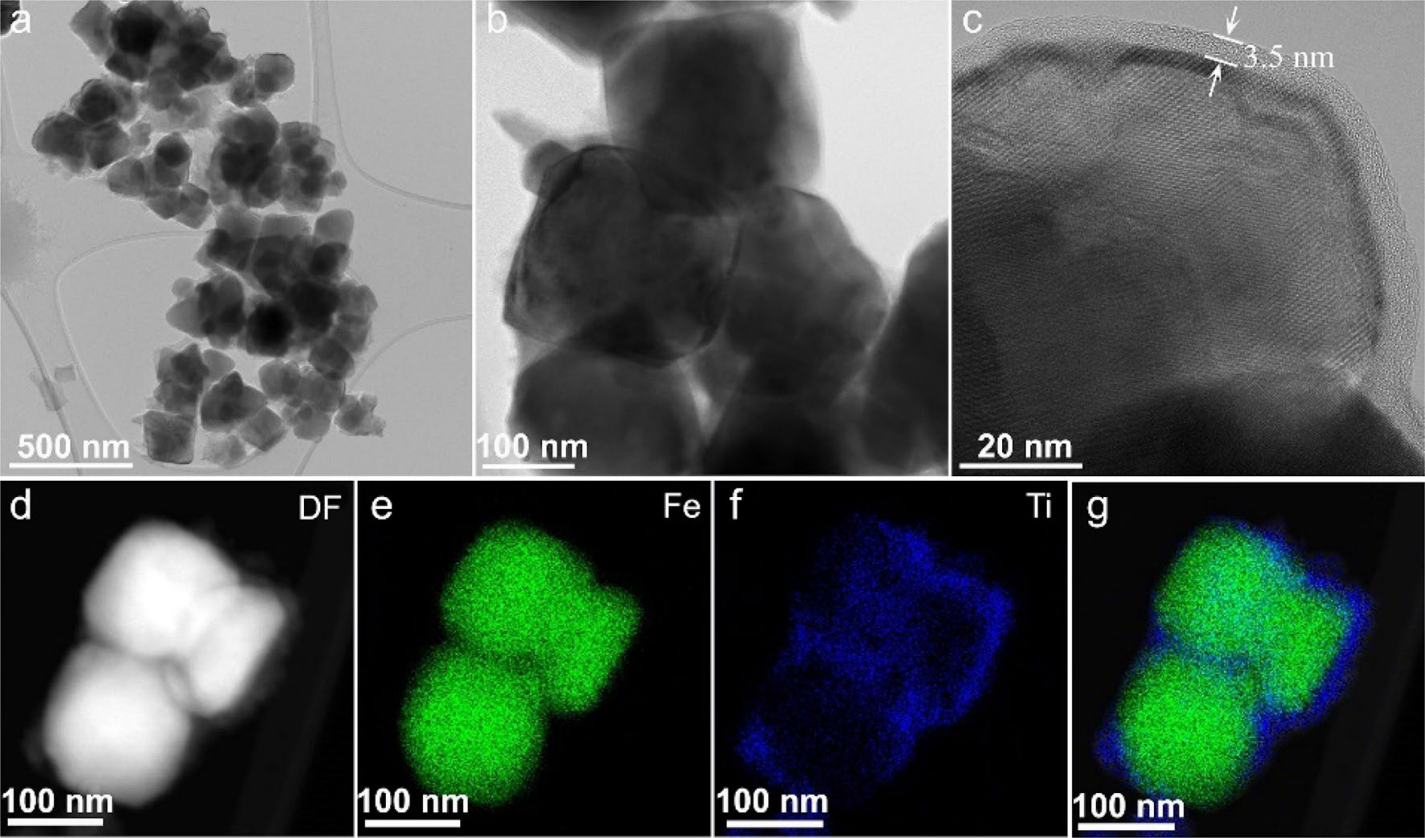
CTEM (**a**, **b**) and HRTEM (**c**) images of SIAL nanocomposites—iron oxide/TBT 500 mg/ml treated at 200 °C (NC-SIAL-500/200); dark field (DF)-STEM image of nanocomposites (**d**), elemental maps of Fe (**e**) and Ti (**f**) and the overlay of the two maps (**g**).

**Figure 4:**
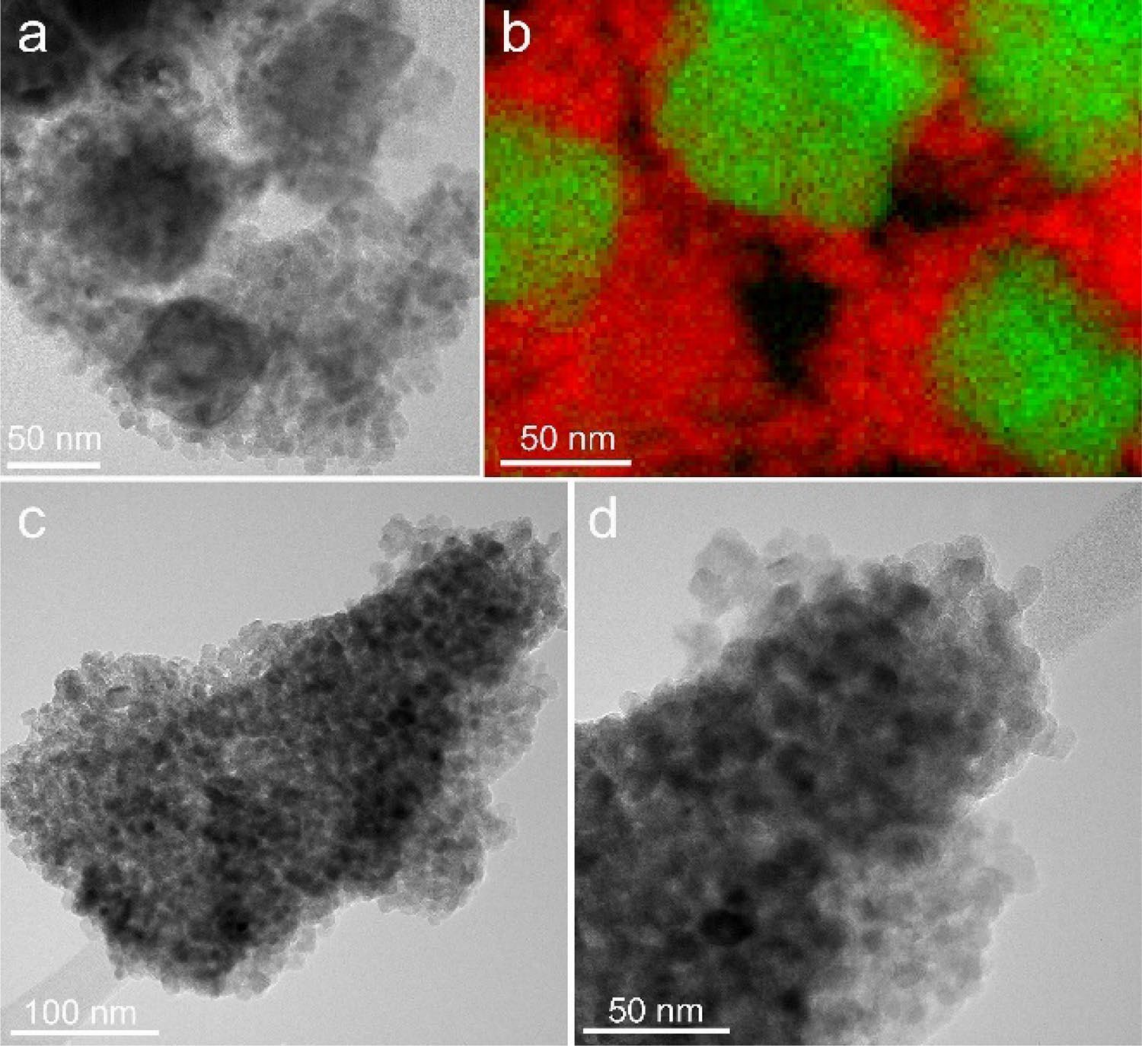
CTEM image (**a**) and overlay (**b**) of EELS elemental maps of Fe (green) and Ti (red) for PP nanocomposites—iron oxide/TBT 200 mg/ml treated at 400 °C (NC-PP-200/400); CTEM images (**c**, **d**) for COPP nanocomposites (NC-COPP-200/400).

The microscopic structure of the produced nanocomposites depended on the iron oxide/TiO_2_ ratio and thermal treatment temperature, as shown by the CTEM, high resolution TEM (HRTEM) and dark field-STEM (DF-STEM) images in Figs. 2 and 3. In all cases, TEM observations revealed smaller or larger agglomerates of IONPs covered or mixed with various amounts of TiO_2_ with different crystallinity. For high Fe/Ti ratios, the larger SIAL and PP nanoparticles formed of core–shell-like structures, as illustrated in Fig. 3. At low thermal treatment temperatures, below 300 °C, the thin TiO_2_ shells surrounding groups of IONPs were homogeneous, continuous, amorphous or little crystallized, with thickness in the range 3–10 nm. The HRTEM image in Fig. 3c shows an example of a thin amorphous shell with size of 3.8–5.5 nm. The EDX elemental mapping (Fig. 3e–g) confirmed the core–shell relative distribution of the iron and titanium phases. When the Fe/Ti ratio was low, the obtained nanocomposites were solid dispersions of IONPs embedded in TiO_2_ matrices (Fig. 4). For thermal treatment temperatures beyond 350 °C, water loss and crystallization processes induced the formation of heterogeneous and discontinuous TiO_2_ matrices consisting of aggregates of crystalized TiO_2_ nanoparticles with sizes ranging from 3 to 9 nm (Fig. 4a). The EELS elemental maps in Fig. 4b shows the discontinuous TiO_2_ nanoparticle aggregate matrix (red) surrounding the PP iron oxide nanoparticles (green).

In the case of small size COPP IONPs, the size of the iron phase was comparable to the size of the crystalized TiO_2_ nanoparticles and the microscopic structure of the obtained nanocomposites resembled neither a core–shell nor a dispersed-matrix pattern but rather consisted of randomly mixed aggregates of TiO_2_ and iron oxide nanoparticles (Fig. 4c,d). No significant differences with respect to the size and morphology of the crystalized TiO_2_ nanoparticles were observed between the nanocomposite samples.

The evolution of crystal phases in the produced nanocomposites (NC) was investigated by separately studying the behavior of IONPs, NC and TiO_2_ following the thermal treatment (TT). In regard to the iron phases, the increase of TT temperature led to the formation of higher amounts of γ-Fe_2_O_3_ in all the IONPs samples. At temperatures of 400 °C and higher, the occurrence of the non-magnetic α-Fe_2_O_3_ phase was revealed by the XRD determinations.

**Figure 5:**
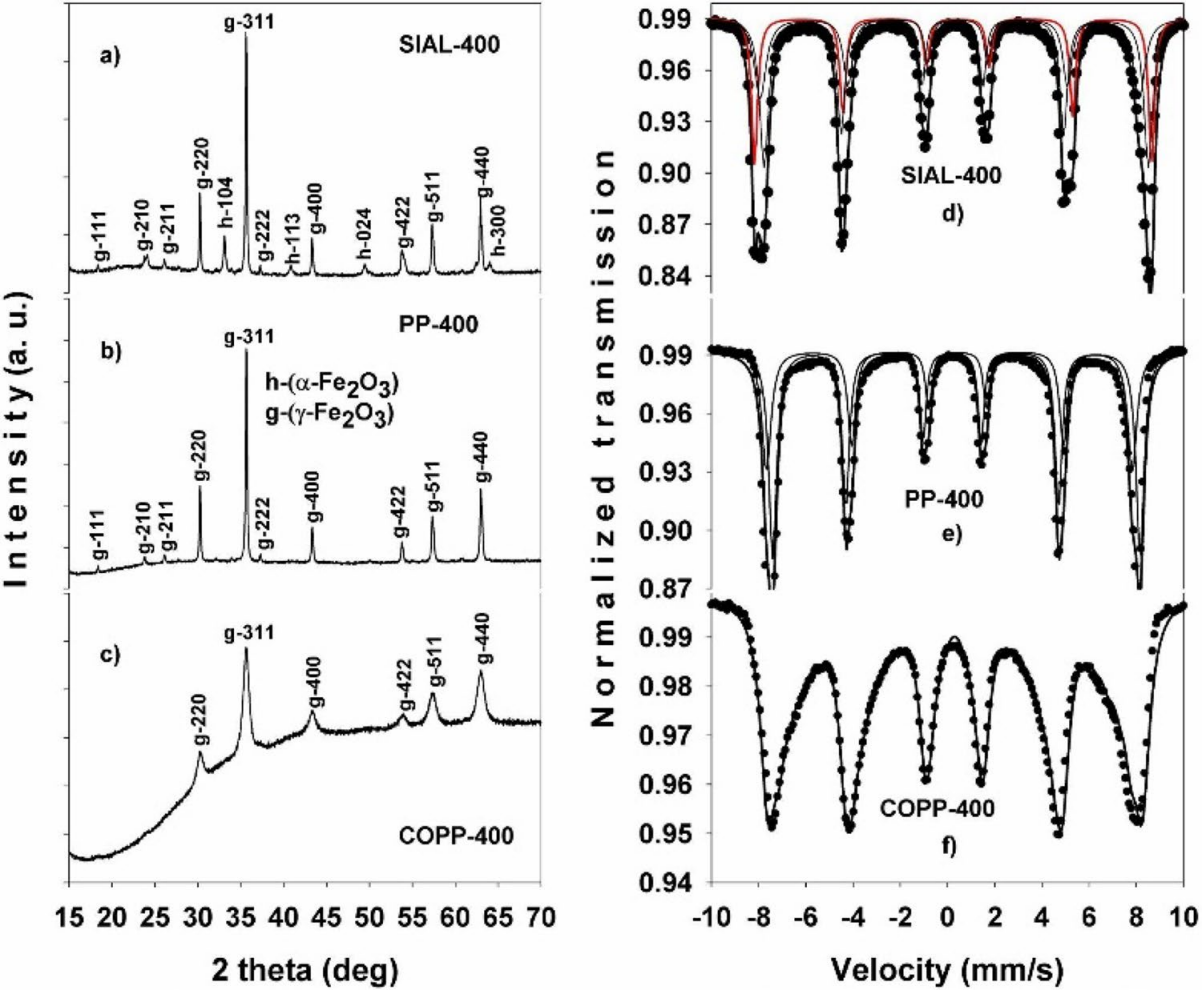
X-ray diffractograms (**a**–**c**) and fitted Mössbauer spectra (**d**–**f**) of the initial iron oxides treated at 400 °C; the continuous red line represents the contribution from the α-Fe_2_O_3_ phase.

**Figure 6:**
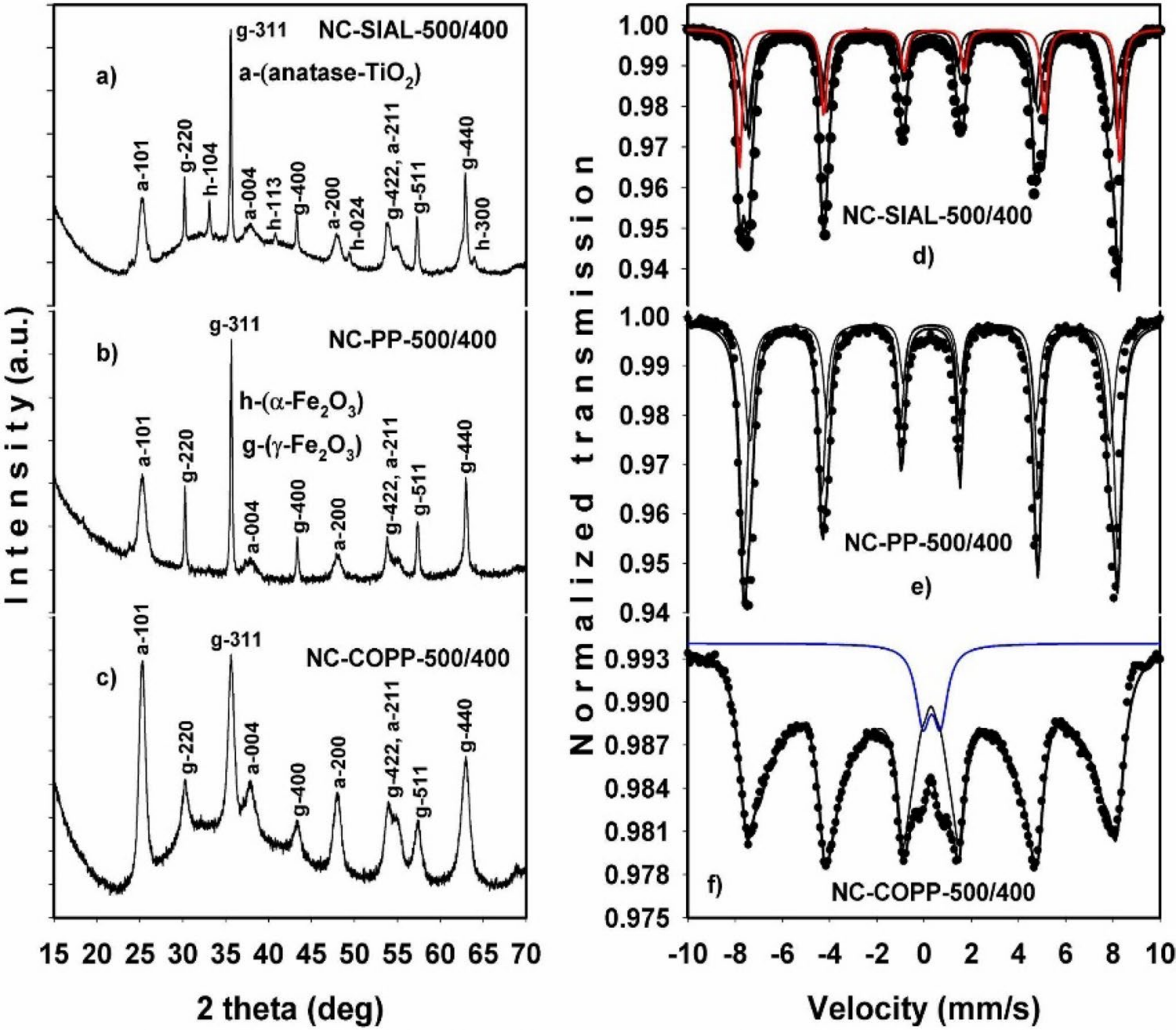
X-ray diffractograms (**a**–**c**) and Mössbauer spectra (**d**–**f**) of nanocomposites (500 mg iron oxide/ml TBT) produced at 400 °C; the continuous red line represents the contribution from the α-Fe_2_O_3_ phase; the continuous blue line represents the superparamagnetic doublet.

The XRD and Mössbauer spectroscopy results for the IONPs and NC treated at 400 °C are displayed in Figs. 5 and 6, respectively. At this temperature, the phase composition of the initial iron oxide samples, as revealed by the Rietveld refinement of the XRD data, became: SIAL (75% γ-Fe_2_O_3_ and 25% α-Fe_2_O_3_), PP (100% γ-Fe_2_O_3_), and COPP (100% γ-Fe_2_O_3_). The information extracted from the fitted Mössbauer spectra of the initial IONPs treated at 400 °C is summarized in Table 2. In the case of the SIAL-400 sample, the results revealed the presence of two iron phases, maghemite (γ-Fe_2_O_3_) and hematite (α-Fe_2_O_3_), with the relative abundance of 72% (γ-Fe_2_O_3_) and 28% (α-Fe_2_O_3_) (continuous red line in Fig. 5d), respectively. The spectrum of the PP-400 sample (Fig. 5e) contained only the characteristic two magnetic sublattices of the maghemite phase. In the case of the COPP-400 sample, its spectrum could only be fitted with a magnetic hyperfine field distribution, being again collapsed due to the small particle size effect (Fig. 5f).

**Table 2:** Mössbauer fit results for the initial iron oxides treated at 400 °C.

Sample	IS* (mm/s)	∆E_Q_ (mm/s)	H_hf_ (T)	Site assignment/area (%)	Relative abundance (%)
SIAL-400	0.345	0.162	50.3	Maghemite (B)/42	72
	0.323	− 0.337	49.6	Maghemite (A)/30	
	0.403	− 0.198	51.9	Hematite/28	28
PP-400	0.413	− 0.133	51.5	Maghemite (B)/61	100
	0.348	− 0.070	49.7	Maghemite (A)/39	
COPP-400	0.381	0.026	15.8–48.6	Hyperfine fields distribution (100)	100
Errors	± 0.002	± 0.004	± 0.03		± 1.0

In the case of nanocomposites, Fig. 6 illustrates the results obtained for the samples with the highest Fe/Ti ratio (500 mg IONPs/ml TBT). Due to the presence of a significant amount of anatase TiO_2_ in the analyzed samples, as revealed by XRD (Fig. 6a–c), the iron phases were best characterized by MS (Fig. 6d–f).

The Mössbauer spectrum of the NC-SIAL-500/400 sample revealed an iron phase composition consisting of 74% γ-Fe_2_O_3_ and 26% α-Fe_2_O_3_, values close to those obtained for the SIAL-400 sample (Table 3). The situation was similar in the case of the NC-PP-500/400 composite sample, with no significant differences regarding the hyperfine parameters and the relative abundance of iron phases being observed as compared with the PP-400 sample. In the case of the NC-COPP-500/400 composite, the spectrum was best deconvoluted in a magnetic hyperfine field distribution (as in the case of COPP-400) accompanied by a central quadrupole pattern with large line widths (~ 0.72 mm/s) and an area of 7% of the total spectrum. With an isomer shift of ~ 0.39 mm/s and ΔE_Q_ of ~ 0.74 mm/s, this doublet suggests the contribution to the Mössbauer spectrum from the Fe^3+^ ions located in the smallest (superparamagnetic) particles of the sample (< 5 nm)^51,52^.

**Table 3:** Mössbauer fit results for the nanocomposites (500 mg iron oxide/ml TBT) produced at 400 °C.

Sample	IS* (mm/s)	ΔE_Q_ (mm/s)	H_hf_ (T)	Site assignment/area (%)	Relative abundance (%)
NC-SIAL-500/400	0.384	0.171	50.5	Maghemite (B)/45	74
	0.341	− 0.394	49.8	Maghemite (A)/29	
	0.412	− 0.218	52.1	Hematite/26	26
NC-PP-500/400	0.399	0.025	50.9	Maghemite (B)/62	100
	0.368	− 0.079	48.9	Maghemite (A)/38	
NC-COPP-500/400	0.378	0.027	9.0–48.2	Hyperfine fields distribution/93	93
	0.394	0.737	–	Superparamagnetic doublet/7	7
Errors	± 0.002	± 0.004	± 0.03		± 1.0

**Figure 7:**
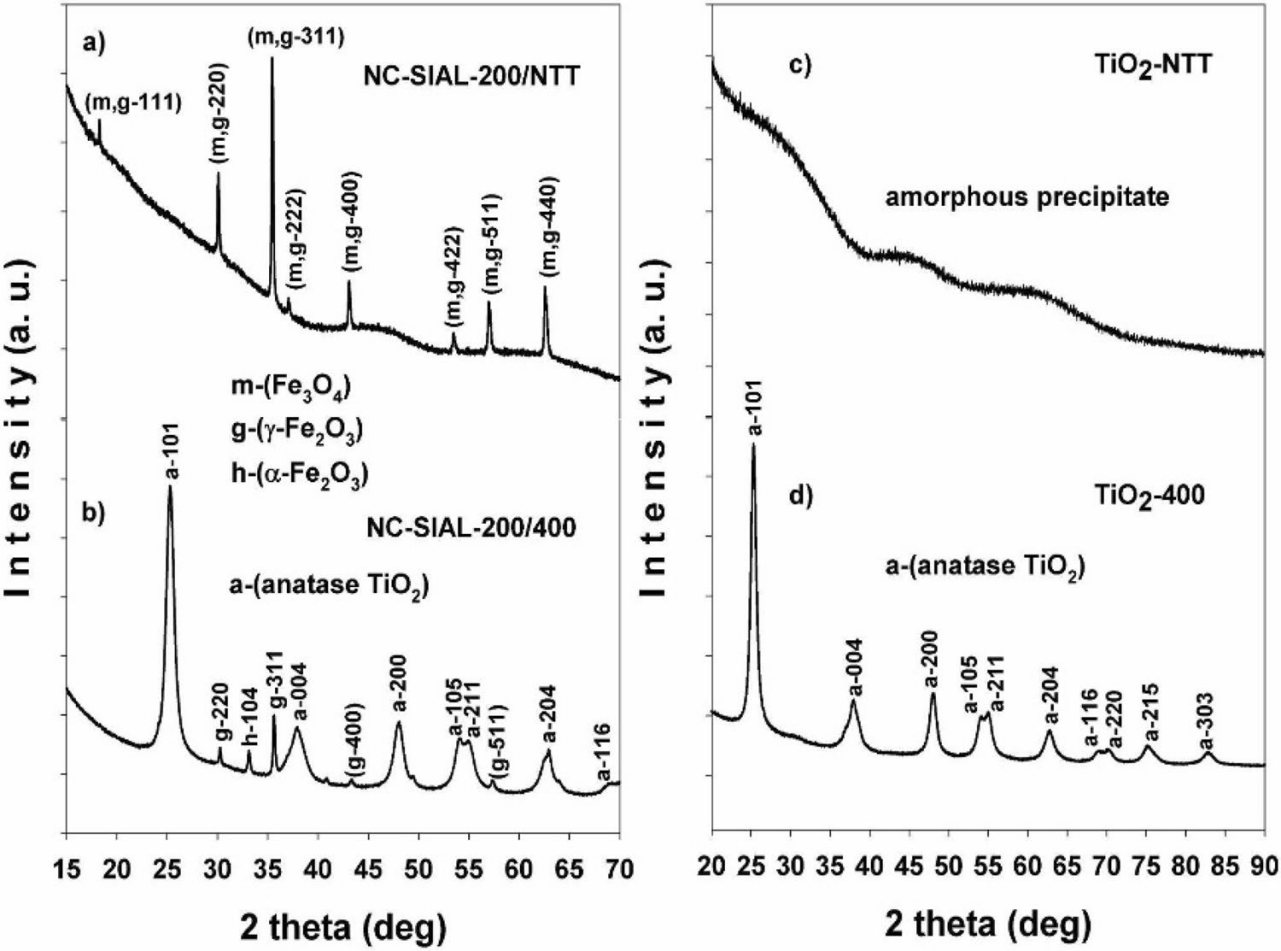
X-ray diffractograms of: (**a**) not thermally treated (NTT) SIAL-based nanocomposite (200 mg SIAL iron oxide/ml TBT) (NC-SIAL-200/NTT); (**b**) nanocomposite treated at 400 °C (NC-SIAL-200/400); (**c**) TiO_2_ precipitate without thermal treatment (TiO_2_-NTT); (**d**) TiO_2_ precipitate treated at 400 °C (TiO_2_-400).

In order to assess the thermal evolution of the titanium phases and the possible influence of the iron oxide presence upon the TiO_2_ crystallization, the XRD results for the NC with the Fe/Ti ratio of 200 mg IONPs/ml TBT were compared to those for TiO_2_ alone. Representative X-ray diffractograms of the not thermally treated (NTT) nanocomposite based on the SIAL iron oxide (NC-SIAL-200/NTT) and the titanium precipitate (TiO_2_-NTT) are shown in Fig. 7a and c, respectively. At 400 °C, both samples (Fig. 7b,d) contained anatase TiO_2_ as a single titanium phase.

**Figure 8:**
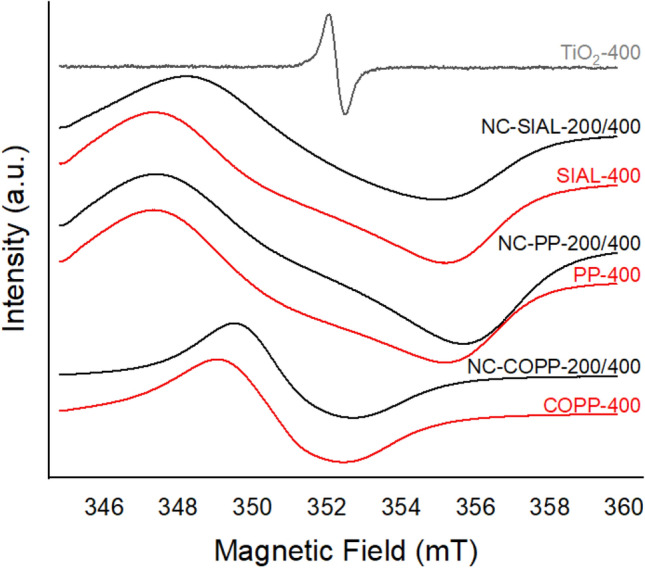
EPR spectra of samples treated at 400 °C: TiO_2_ precipitate (gray), nanocomposites (black) and the corresponding IONPs (red).

The EPR spectra of the TiO_2_ precipitate treated at 400 °C (TiO_2_-400), IONPs treated at 400 °C, and of the corresponding nanocomposite samples are illustrated in Fig. 8. The recorded iron oxide spectra were very broad due to the ferromagnetic character of iron, which influenced the applied external magnetic field. The addition of TiO_2_ influenced the EPR spectra of the iron oxide samples by slightly changing the peak-to-peak line width, thus indicating a small change in the ligand field of the paramagnetic species contained by the nanocomposites. However, due to the ferromagnetic effects of iron, all EPR spectra were broadened and most of the contained information was lost due to the extensive line width.

**Figure 9:**
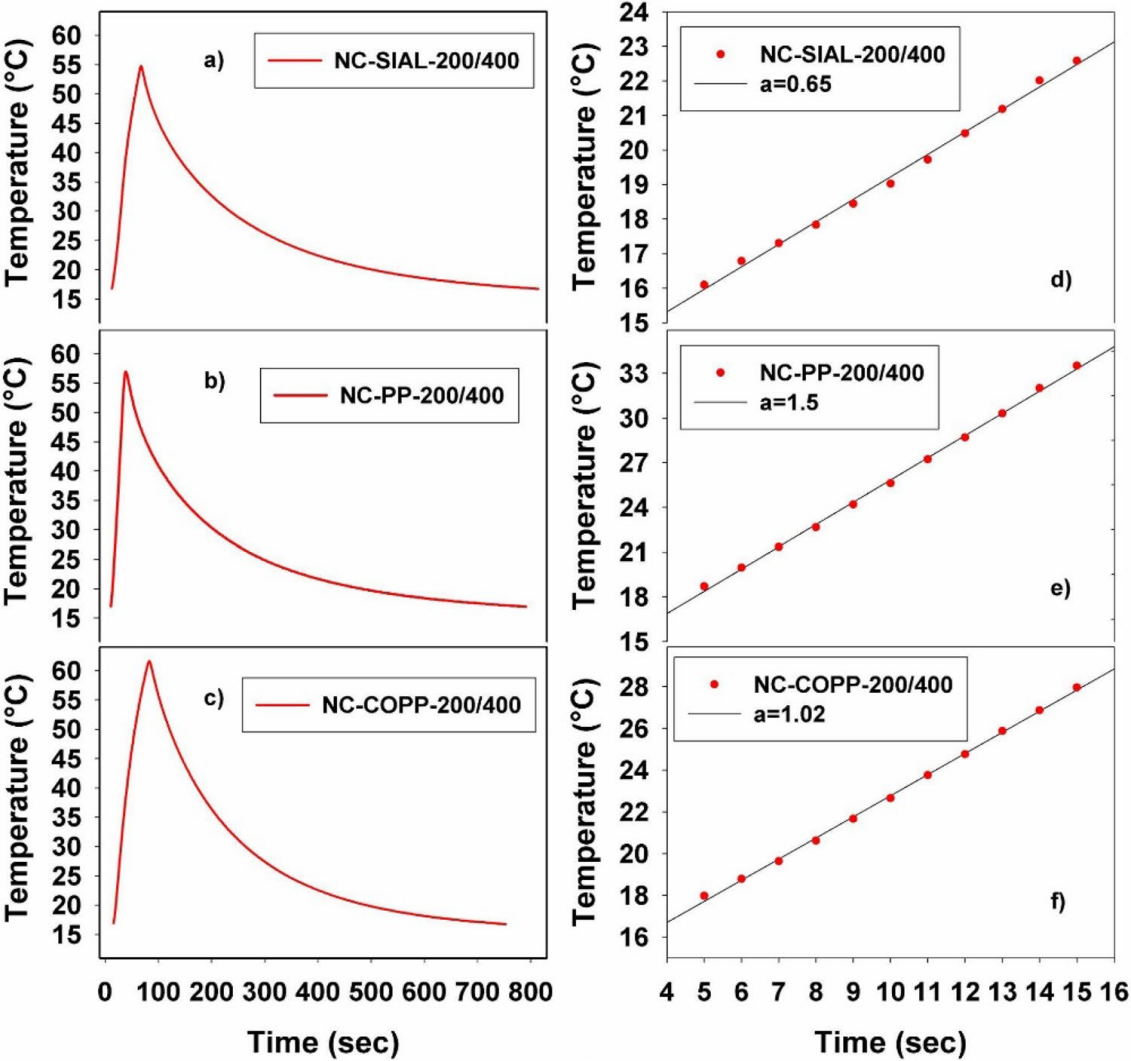
Heating–cooling curves (**a**–**c**) and linear fits with slopes “a” (**d**–**f**) for nanocomposites (200 mg iron oxide/ml TBT synthesized at 400 °C) exposed to an alternating magnetic field of 175 Oe.

The efficiency of AC magnetic heating of the synthesized nanocomposites was determined based on the temperature curves, T(t), recorded for different intensities of the applied magnetic field. Examples of time-dependent heating–cooling curves recorded for SIAL nanocomposites exposed to an AC magnetic field of 175 Oe are displayed in Fig. 9a–c. The heating rates (HRs) were determined based on the slopes of the regression lines obtained by fitting the linear part of the heating curves, as shown in Fig. 9d–f. For the illustrated case, the HR values were 0.96 °C/s, 1.6 °C/s, and 0.99 °C/s for the NC-SIAL-200/400, NC-PP-200/400, and NC-COPP-200/400 samples, respectively. The heating efficiency of the NC-PP-200/400 sample was higher compared to those of the NC-SIAL-200/400 and NC-COPP-200/400 nanocomposites for all the tested field intensities.

The obtained heating rates were further used to determine the specific absorption rates (SAR_IONPs_) of the tested samples. The specific heat capacity of the magnetic fluids was approximated, using the formula given in the Introduction section, at c = 3.86 J g^−1^ K^−1^. This approximate value was obtained as further described bellow. Since the values of specific heat for water and PEG-1000 are 4.18 J g^−1^ K^−1^ and 0.26 J g^−1^ K^−1^, respectively, a value of 3.72 J g^−1^ K^−1^ results for the used PEG-1000 solution (25% PEG-1000 in water). Taking into account the specific heats of the nanocomposite components (0.68 J g^−1^ K^−1^ for TiO_2_ and 0.64 J g^−1^ K^−1^ for Fe_3_O_4_) and a magnetic volume fraction of 0.02, the specific heat of the nanocomposite suspension becomes 3.86 J g^−1^ K^−1^, being only slightly different from that of the dispersion medium. The weigth content of the samples corresponds to 0.46 mg of IONPs and 0.54 mg of TiO_2_ per mg of nanocomposite, resulting a mass of 0.1 g of IONPs in each sample. Similar specific heats have been considered for the iron phases present in the nanocomposite samples. This approximation is acceptable since the specific heat of the magnetic component has only a tiny contribution to the specific heat of the nanocomposite suspension. The highest heating efficiency was found in the case of the PP iron oxide-based nanocomposites.

**Figure 10:**
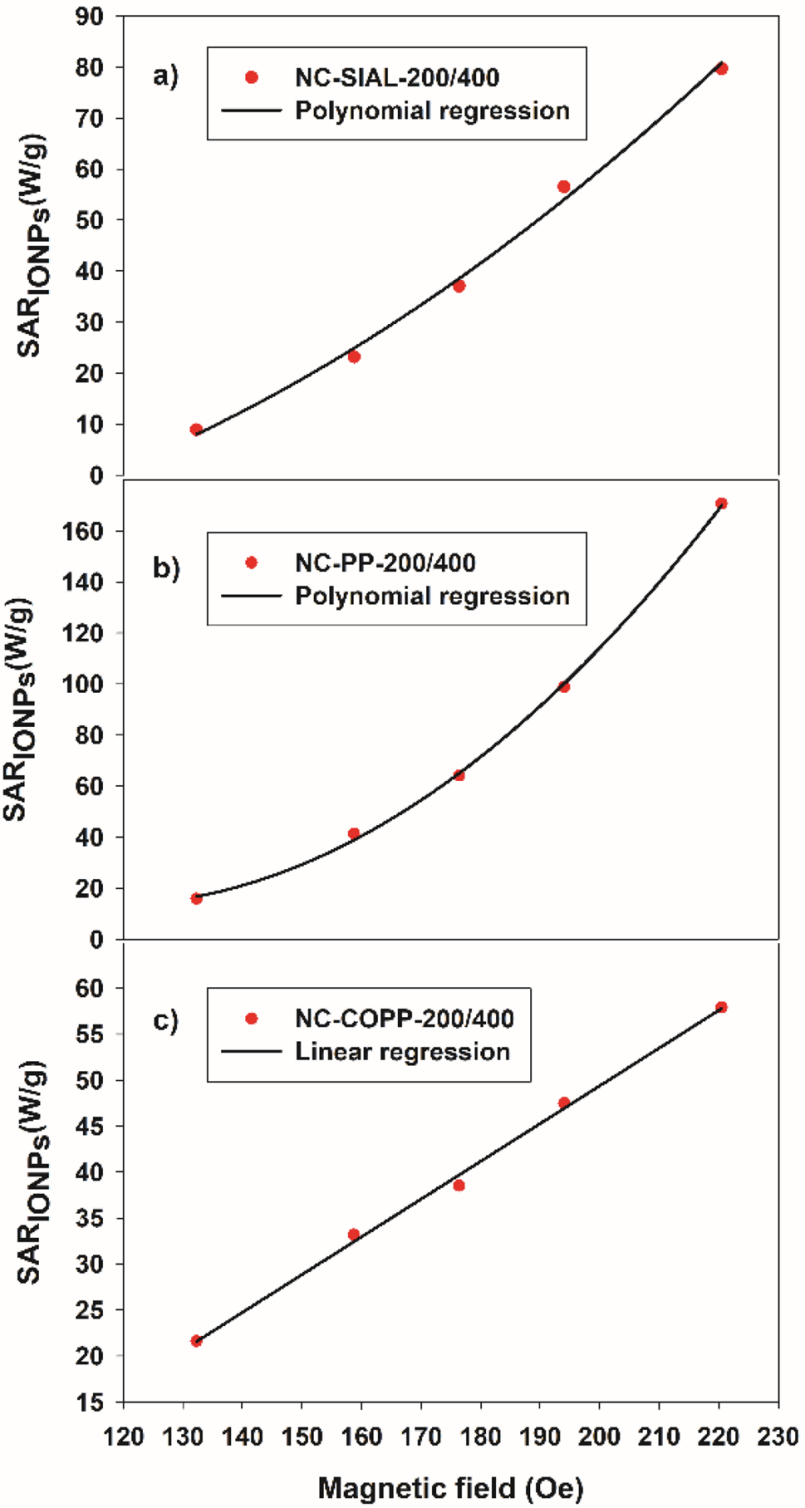
Specific absorption rate (SAR) dependence on magnetic field intensity for nanocomposites (200 mg iron oxide/ml TBT synthesized at 400 °C).

The dependence of the determined SAR on the intensity of the applied magnetic field is illustrated in Fig. 10a–c. A second-order polynomial dependence was observed in the case of the NC-SIAL-200/400 and NC-PP-200/400 samples and a linear dependence in the case of NC-COPP-200/400.

The efficiency of ROS photogeneration by TiO_2_ alone and by the synthesized iron oxide-TiO_2_ nanocomposites was assessed by EPR using the spin trapping agent 5,5-Dimethyl-1-Pyrroline-N-Oxide (DMPO). DMPO can trap oxygen radicals produced after the photoexcitation of TiO_2_^16–18^, collectively denoted by ·R, forming more stable adducts DMPO + ·R → ·DMPO–R. The nature of the trapped radicals can be identified by analyzing the experimental EPR spectra^53^.

**Figure 11:**
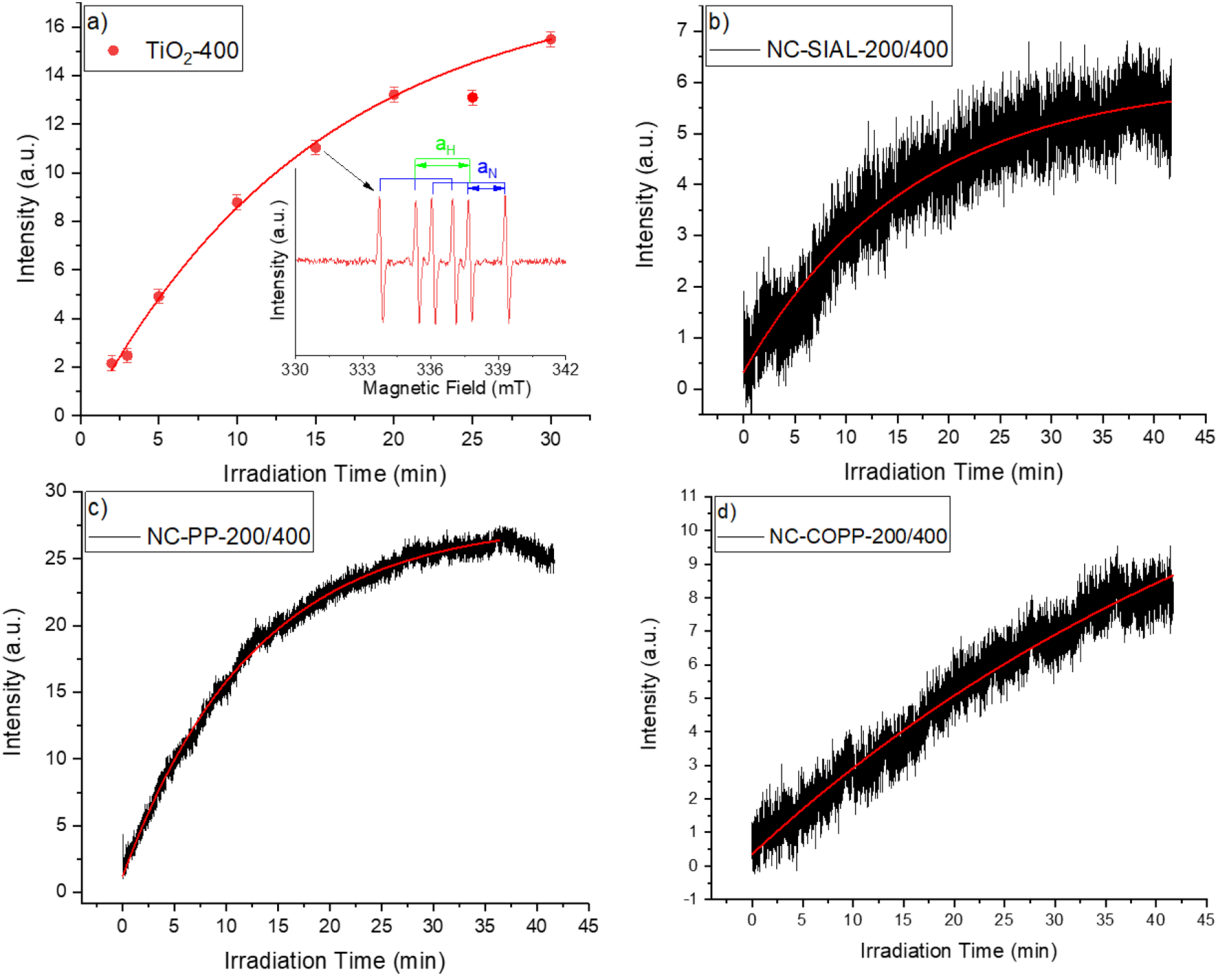
ROS photogeneration kinetics (UV 365 nm) for: (**a**) TiO_2_-400 (inset: EPR spectrum); (**b**) NC-SIAL-200/400; (**c**) NC-PP-200/400; (**d**) NC-COPP-200/400.

The inset of Fig. 11a depicts the six-equal line EPR spectrum recorded immediately following the UV irradiation (365 nm) of the titanium precipitate treated at 400 °C (TiO_2_-400). The hyperfine constants determined from the spectrum were a_N_ = 16.3 G and a_H_ = 23.3 G. The evolution of the EPR signal intensity for the UV-irradiated TiO_2_-400 sample is represented as a function of irradiation time in Fig. 11a. The curve was well fitted by a mono-exponential model and the reaction constant, K_TiO2_ = 0.07 min^−1^, was determined. The kinetics of ROS photogeneration by the NC-SIAL-200/400, NC-PP-200/400 and NC-COPP-200/400 nanocomposite samples are represented in Fig. 11b–d, the following reaction constants being calculated by mono-exponential fitting of the kinetics curves: K_NC-SIAL-200/400_ = 0.06 min^−1^, K_NC-PP-200/400_ = 0.08 min^−1^ and K_NC-COPP-200/400_ = 0.017 min^−1^.

The adsorption of bovine serum albumin from cell culture medium onto the nanocomposites with the highest ROS photogeneration activity was studied by FTIR spectroscopy.

**Figure 12:**
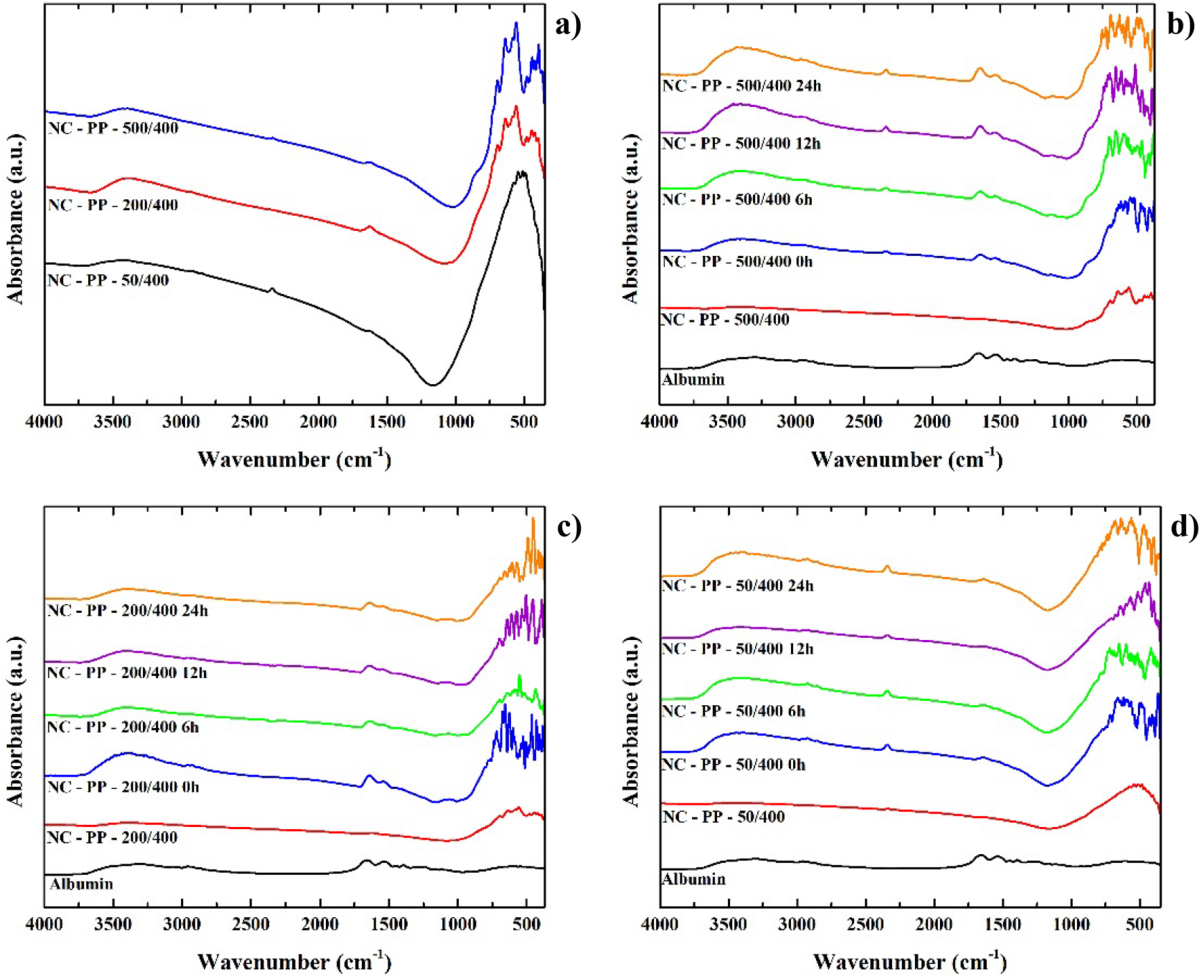
Infrared spectra of the NC-PP-500/400, NC-PP-200/400 and NC-PP-50/400 nanocomposite samples: (**a**) comparison between the as-synthesized NCs; (**b**–**d**) comparison between NCs ultrasonicated in supplemented DMEM for 6 h and further incubated in supplemented DMEM for 0 h, 6 h, 12 h and 24 h.

The infrared spectra of the three nanocomposites obtained using the PP iron oxide are presented in Fig. 12a. The sample with the smallest Fe/Ti ratio, NC-PP-50/400, presents absorption bands characteristic to TiO_2_ in the 900–500 cm^−1^ spectral range^54^ and a low-intensity absorption band at ~ 2900 cm^−1^ which corresponds to the stretching vibration mode of –C–H bonds. The presence of these bonds is likely to originate from the titanium butoxide precursor used in the synthesis of the nanocomposites. Another weak absorption band can be observed at ~ 2360 cm^−1^, in the case of the NC-PP-50/400 and NC-PP-500/400 samples being more visible, which can be assigned to –C=O vibration mode from CO_2_ absorbed from the air^55^.

Besides the presence of TiO_2_, the spectra of NC-PP-200/400 and NC-PP-500/400 reveal their higher iron oxide content with proportional intensity characteristic absorption bands in the 900–450 cm^−1^ range^56^.

The adsorption of serum albumin from supplemented DMEM was studied with respect to time and chemical environment used to disperse the nanocomposites prior to the incubation with the culture medium. The occurrence and time-dependence of the adsorption process in the case of samples ultrasonicated in supplemented DMEM are illustrated in Fig. 12b–d. The bands corresponding to the vibration modes of albumin are visible in all cases and their presence indicates that the albumin adsorption takes place even during the ultrasonication stage (0 h of incubation with supplemented DMEM). The process shows a weak incubation time-dependence, more prominent in the case of NC-PP-500/400. A negative correlation with the amount of TiO_2_ in the samples was also observed, the infrared absorption bands of albumin being more intense in the case of the NC-PP-500/400 sample compared to NC-PP-50/400 and NC-PP-200/400, respectively.

The IR spectrum of albumin (shown in Fig. 12b–d) consists of absorption bands which are characteristic of stretching vibration modes of –O–H and –N–H bonds from the amide A group in the 3000–3400 cm^−1^ spectral range. In the 2800–3050 cm^−1^ there are the absorption bands characteristic to the stretching vibration modes for –C–H bonds and at ~ 1650 cm^−1^ for the amide I group which mainly consists of –C=O stretching vibrations modes. Around 1540 cm^−1^ occur the absorption bands characteristic to the bending vibration mode of –N–H bonds from the amide II group, which are vibrations of –C–N–H angles^57^. All of the absorption bands of albumin are found in the infrared spectra of the nanocomposites, only a slight shifting of ~ 15 cm^−1^ in the maximum of the absorption band characteristic to the –C=O stretching vibration mode of the amide I group being observed. This shift to lower wavenumbers suggests that albumin is adsorbed onto the surface of the nanocomposites through the amide I group which mainly consists of –C=O bonds, most probably through hydrogen bonding, and could also indicate conformational changes in the BSA secondary structure^58,59^.

We further discuss the way in which the adsorption of albumin during incubation with supplemented culture medium is influenced when nanocomposites are previously dispersed in commonly used buffers: HBSS and NaCl solution. Given the previous observations (weak incubation time-dependence and maximum adsorption at 24 h of incubation), the study was performed on samples ultrasonicated in HBSS or NaCl solution which were not incubated with supplemented DMEM afterward (0 h of incubation) and on samples incubated for 24 h with supplemented DMEM following ultrasonication.

**Figure 13:**
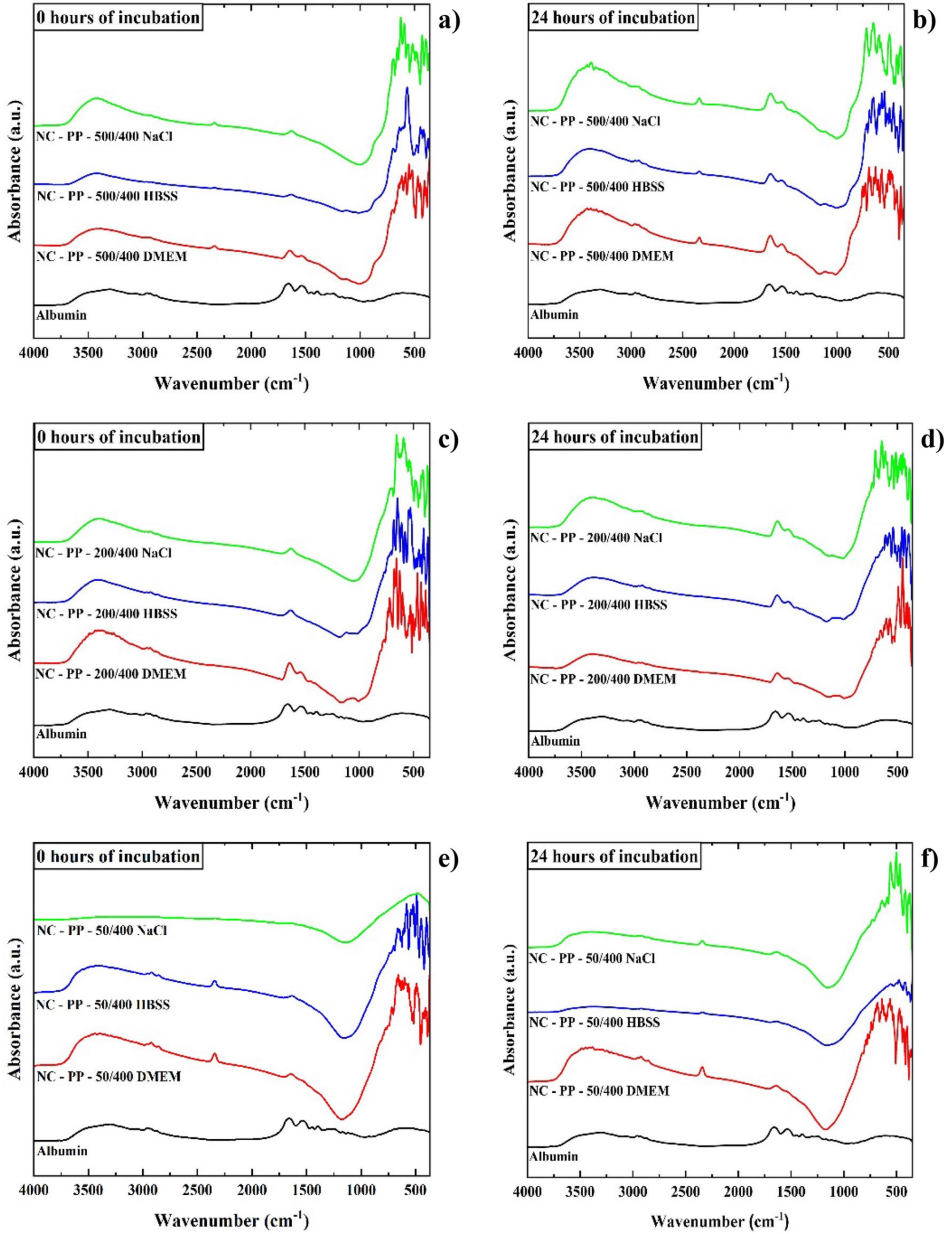
Infrared spectra of the nanocomposites NC-PP-500/400, NC-PP-200/400 and NC-PP-50/400: (**a**, **c**, **e**) ultrasonicated in NaCl solution, HBSS and supplemented DMEM (0 h of incubation in culture medium) and (**b**, **d**, **f**) incubated for 24 h in supplemented DMEM following the ultrasonication.

The infrared spectra recorded for these samples were compared to those of the samples ultrasonicated in supplemented DMEM and further incubated with supplemented DMEM (24 h), the results being presented in Fig. 13a–f.

The results illustrated in Fig. 13 show that in the case of the NC-PP-50/400 sample, having the lowest Fe/Ti ratio (highest amount of TiO_2_), albumin adsorption was minimal and virtually independent of the environment in which the ultrasonication was performed. The adsorption was more efficient for the samples ultrasonicated in supplemented DMEM, compared to those ultrasonicated in HBSS and NaCl solution. The albumin adsorption was most prominent in the case of the NC-PP-500/400 sample. The results also indicate that ultrasonication in NaCl solution has a very slight enhancement effect upon albumin adsorption onto the nanocomposites compared to HBSS.

**Figure 14:**
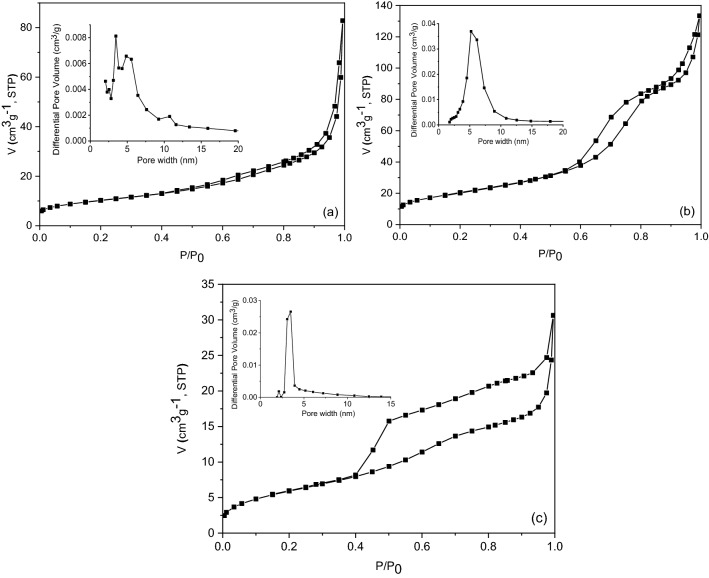
N_2_ adsorption–desorption isotherms and pore size distributions (inset of the figures) of the investigated samples: (**a**) NC-PP-500/400; (**b**) NC-PP-200/400; (**c**) NC-PP-50/400.

The textural features of the samples were investigated by nitrogen physisorption measurements. All isotherms (Fig. 14) are of type IV^60^, typical for mesoporous materials, with H3 hysteresis loops. The textural parameters (BET surface area, total pore volume and average pore diameters) are listed in Table 4. Among the investigated samples, NC-PP-200/400 showed the highest values for BET surface area and total pore volume, followed by NC-PP-500/400 and NC-PP-50/400. This result can be explained considering the formation of compact aggregates during the synthesis process as follows: (i) clusters composed of several IONPs covered altogether by a thin shell of TiO_2_ in the case of NC-PP-500/400 (Fig. 3) and (ii) clusters composed of a larger number of small TiO_2_ nanoparticles in the case of NC-PP-50/400. This clustering process led to a reduction of the surface area and pore volume. In the case of NC-PP-200/400, the used Fe/Ti ratio allowed a more efficient separation of the IONPs (Fig. 4) and led to a reduction of the degree of compactness (higher porosity) of the TiO_2_ clusters.

**Table 4:** Textural parameters and zeta potential of the nanocomposite samples.

Sample	S_BET_ (m^2^ g^−1^)	V_total_ (cm^3^ g^−1^)	Average pore diameter (nm)	Zeta potential (mV)
NC-PP-500/400	36.0	0.128	13.4	− 7.7
NC-PP-200/400	74.0	0.206	8.3	− 3.1
NC-PP-50/400	22.0	0.047	5.5	− 7.6

Analyzing the pore size distribution graphs, one can notice that NC-PP-500/400 displays a multimodal pore size distribution in a range wider than in the case of NC-PP-200/400 and NC-PP-50/400. This could also be attributed to the presence of aggregates of IONPs of various sizes, covered/mixed with TiO_2_, which generate different pore sizes. The average pore size diameters (Table 4) show a decreasing tendency with the decrease of Fe/Ti ratio, which means that the use of lower concentrations of iron oxides results in a more orderly arrangement of the pores. The values of the textural parameters determined for the investigated samples are comparable to those obtained by other authors for similar materials^61–63^.

The zeta potential values were all negative and small, indicating low electrostatic potential and weak repulsion at the hydrodynamic shear plane. However, one must consider that the tested samples were first dispersed by ultrasonication in FBS supplemented DMEM and the measurements were afterwards performed in the same culture medium, most likely after the formation of the protein corona. It has already been shown that adsorption of BSA onto nanomaterials can lead to the decrease of their zeta potential due to the increase of the distance between the share plane and the particle surface^64^. This implies that, under the experimental conditions used for zeta potential determination, a larger amount of albumin was adsorbed onto the NC-PP-200/400 sample compared to the other two composites. Considering the significantly larger BET surface area and total pore volume of NC-PP-200/400 as well as its average pore diameter (8.3 nm) which is comparable to—or larger than—the hydrodynamic radius of BSA^65^, such an enhanced adsorption is to be expected^66^.

**Figure 15:**
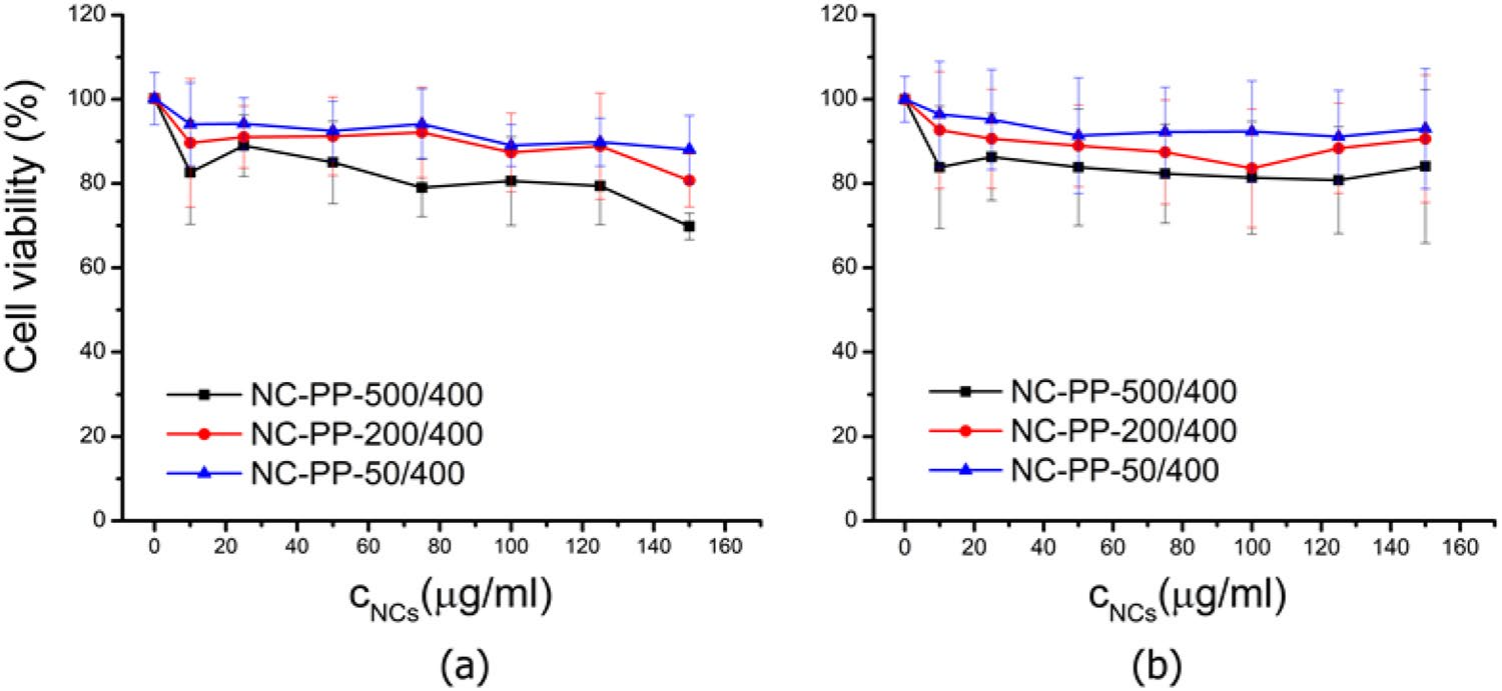
Cell viability of NIH 3T3 cells incubated for 24 h with nanocomposites NC-PP-500/400, NC-PP-200/400 and NC-PP-50/400: (**a**) NCs ultrasonicated in HBSS, (**b**) NCs ultrasonicated in supplemented DMEM. In both cases of ultrasonication the differences were statistically significant (p < 0.05) only for NC-PP-500/400 compared to control for all NCs concentrations.

The biocompatibility of the NCs with the highest hyperthermic and ROS photogeneration activity (i.e., nanocomposites obtained using the PP iron oxide) is further presented. In the case of NIH 3T3, the cell viability was slightly reduced for all categories of NCs, the amplitude of the cytotoxic effect showing a negative correlation with the Fe/Ti ratio. For the NC-PP-500/400 sample, the cell viability was significantly decreased when compared to control (p < 0.05) for all NCs concentrations, irrespective of the ultrasonication procedure. For the highest concentration of NCs (150 µg/ml) ultrasonicated in HBSS (Fig. 15a), the cells’ viability decreased with 30.2% in the case of NC-PP-500/400, 19.3% for NC-PP-200/400 and 11.9% for NC-PP-50/400 when compared to control.

Similarly, for the highest concentration of NCs (150 µg/ml) ultrasonicated in supplemented DMEM (Fig. 15b), the viability decreased with 15.8% in the case of NC-PP-500/400, 9.4% for NC-PP-200/400 and 6.9% for NC-PP-50/400 when compared to control. Within the limits of experimental errors, the observed viability changes were roughly independent of the NCs concentration.

**Figure 16:**
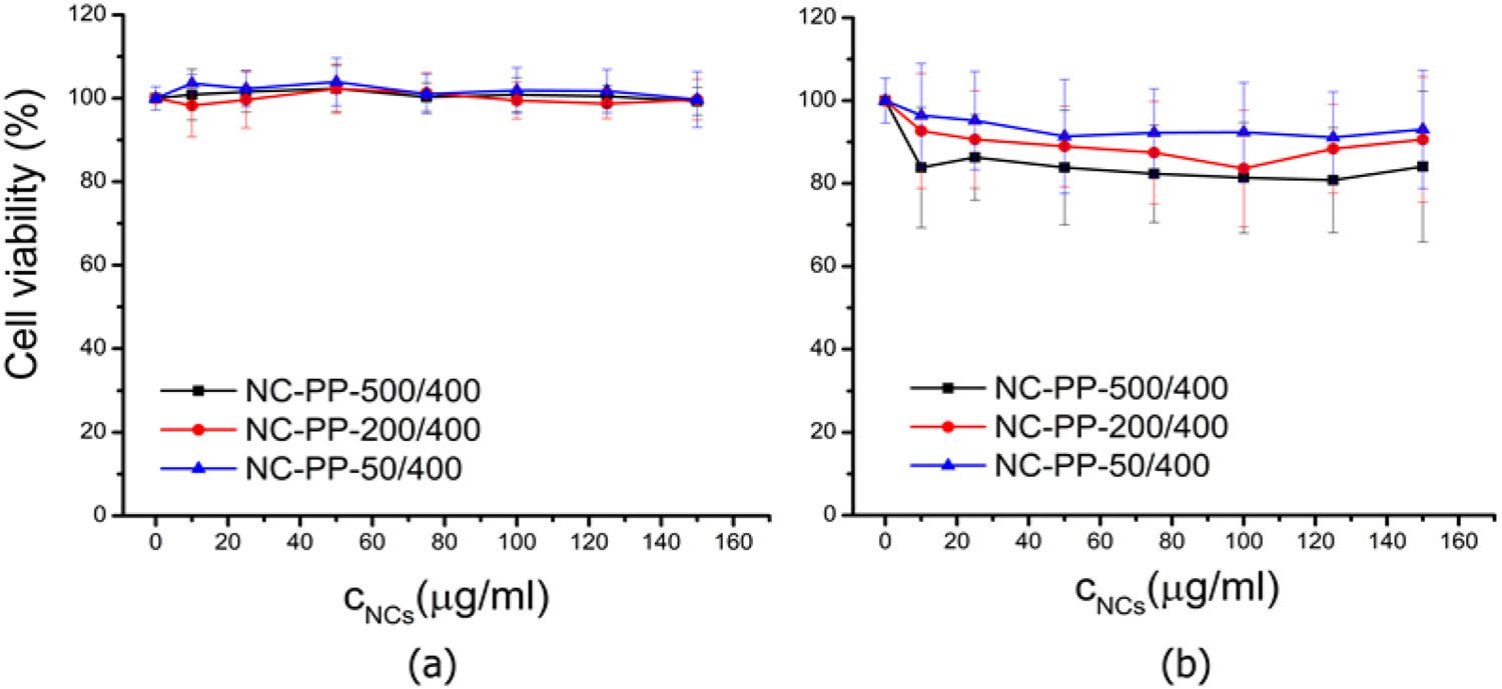
Cell viability of cells incubated for 24 h with nanocomposites (NC-PP-500/400, NC-PP-200/400 and NC-PP-50/400) ultrasonicated in supplemented DMEM: (**a**) HS 27 cells, (**b**) NIH 3T3 cells.

When comparing the human fibroblast cells (HS 27) viability to that of murine fibroblast cells (NIH 3T3) incubated for 24 h with nanocomposites ultrasonicated in supplemented DMEM (Fig. 16), one may see that the two cell lines showed a slightly different response to the NCs. The viability of HS 27 was equal to the control (up to the maximum NC concentration of 150 µg/ml) irrespective of the Fe/Ti ratio.

**Figure 17:**
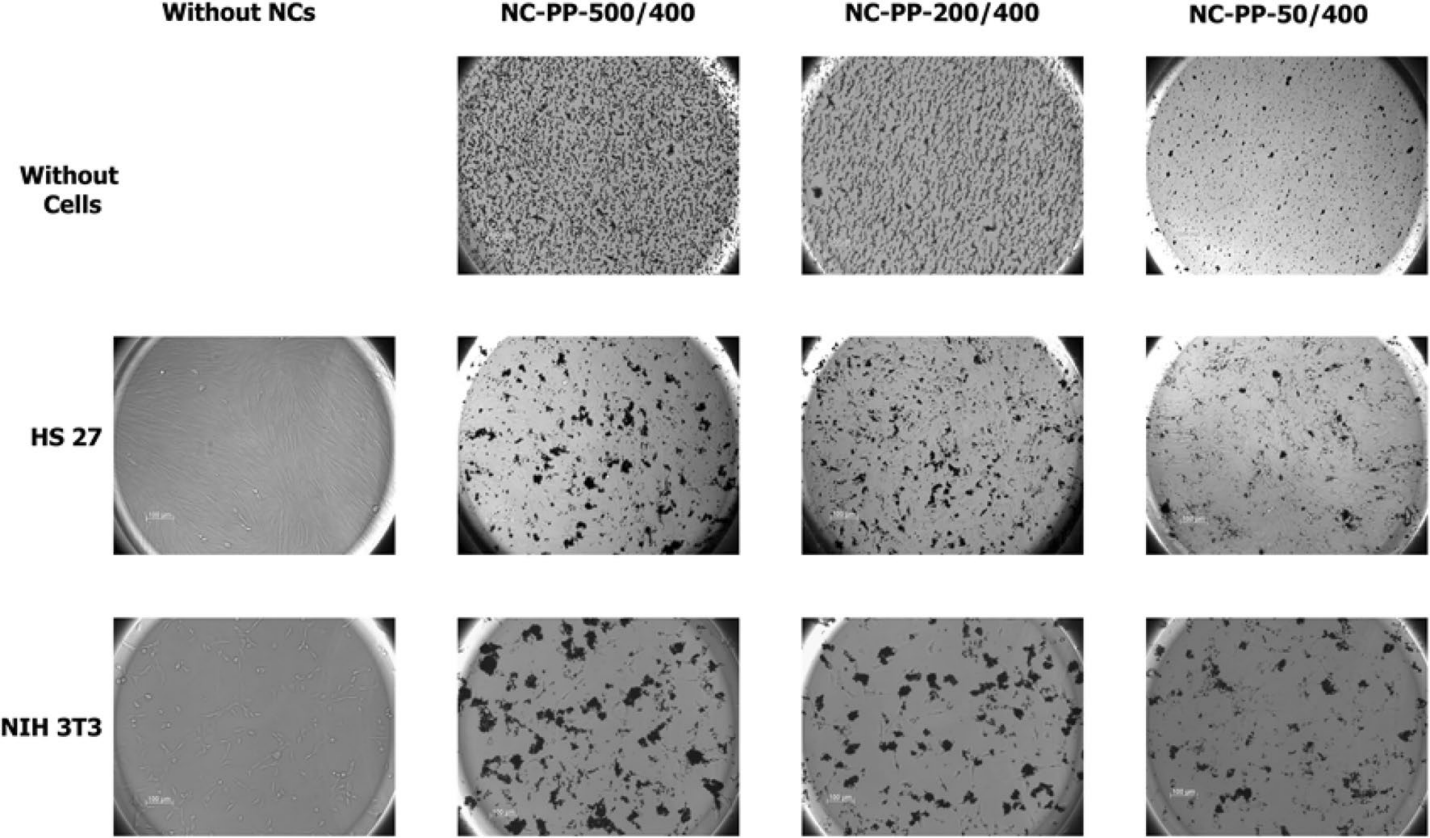
Bright field microscopic images (20 × objective) of NCs suspensions (150 µg/ml) in the absence of cells (top raw) and of cells incubated for 24 h with nanocomposites (NC-PP-500/400, NC-PP-200/400 and NC-PP-50/400) ultrasonicated in supplemented DMEM (NCs final concentration was 100 µg/ml): HS 27 cells (middle raw), NIH 3T3 cells (bottom raw).

In the absence of cells, the NPs distribution in the wells was homogeneous and suggested magnetic interparticle interactions in the case of NC-PP-500/400 and NC-PP-200/400 samples. When the cells were present, there was an agglomeration of nanocomposites on the cell surfaces. This effect was slightly more prominent in the case of NIH 3T3 (Fig. 17, bottom raw) (less NCs were found in spaces between cells in the case of NIH 3T3 compared to HS 27). It was also observed that the higher the Fe/Ti ratio, the higher the clustering.

## Discussion

In the present study we analyze the microscopic structure, phase composition, AC magnetic heating, ROS photogeneration and in vitro cytotoxic behavior of iron oxide-TiO_2_ nanocomposites obtained using three types of initial IONPs: larger ferromagnetic nanoparticles (≈ 100 nm) with similar size distributions and initial phase compositions (SIAL and PP) and superparamagnetic nanoparticles with significantly smaller size (≈ 10 nm) and narrower size distribution (COPP).

When the size of the iron phase nanoparticles is large compared to that of the TiO_2_ phase (≈ 10 nm), the formed nanocomposites have well-defined structure consisting of IONPs surrounded by variable amounts of TiO_2_, depending on the Fe/Ti ratio. When the size of the IONPs is comparable to the size of TiO_2_, the formed nanocomposites consist in aggregates of randomly mixed iron oxide and TiO_2_ nanoparticles, irrespective of the Fe/Ti ratio.

During the thermal treatment of the composites, the TiO_2_ phase is subjected to water loss and crystallization processes and the initial IONPs undergo phase transformations (Fe_3_O_4_ → γ-Fe_2_O_3_ → α-Fe_2_O_3_) with kinetics dictated by their size and initial composition. The iron and titanium phases seem not to have a significant reciprocal influence on their phase transformation and crystallization behavior. While raising the TT temperature, the uniform and continuous amorphous titanium phase observed at low temperatures (below 300 °C, Fig. 4) changes due to dehydration and crystallization processes into a granular and discontinuous structure.

In regard to the iron phases, both Fe_3_O_4_ and γ-Fe_2_O_3_ possess AC magnetic heating properties and are considered nanomaterials with good biocompatibility^11^, being thus appropriate for the purpose of our study. The formation of the weakly ferromagnetic α-Fe_2_O_3_ is to be avoided since its presence reduces the AC magnetic heating capacity of the nanocomposite. This effect is clearly visible in the case of the NC-SIAL-200/400 sample, in which α-Fe_2_O_3_ first occurred, leading to its reduced AC magnetic heating rate (Fig. 9a).

The heating of magnetic fluids is based on the Brownian and Nѐel relaxation processes^3,11^. The Brownian mechanism of relaxation assumes that the magnetic moment is locked to the crystal axis and the particle itself rotates in the fluid when the magnetic moment aligns with the applied field. The Nèel relaxation implies that the magnetic moment is free to rotate (internal rotation) within the crystal. In the case of the synthesized nanocomposites, the physical rotation of the magnetic nanoparticles (and thus the Brownian relaxation mechanism) is either suppressed or hindered by the presence of the surrounding TiO_2_ matrix. This effect is stronger in the case of nanocomposites with low Fe/Ti ratio (larger amount of TiO_2_ in the sample), in comparison to those containing lower amounts of TiO_2_, being composed of smaller size and better dispersed core–shell-like structures. A typical superparamagnetic behavior, as revealed by Mössbauer spectroscopy (Fig. 6f), was observed only in the case of nanocomposites obtained using the small size COPP IONPs.

The EPR determinations evidence the photogeneration of ROS by the obtained iron oxide-TiO_2_ nanocomposites. The expected EPR spectrum of a typical DMPO–OH adduct, formed upon trapping of OH· by DMPO, is composed of a four-line signal with peak height ratios of 1:2:2:1 and associated hyperfine coupling constant a_N_ = a_H_ = 14.9 G^67^. The difference between the observed and expected EPR signals is attributed to the effect of the DMSO in which the DMPO spin trap was dissolved. It has been shown in the literature that DMSO can undergo reactions with hydroxyl radicals OH· to form CH^·^_3_ radicals which are trapped by DMPO, leading to DMPO–CH_3_ adducts with characteristic hyperfine splitting pattern and coupling constants a_N_ = 16.4 G and a_H_ = 23.3 G^68^. These values are very close to those determined from the EPR spectrum of the TiO_2_-400 sample in the present study, providing supporting evidence for the photogeneration of OH· radicals by the studied materials. The differences in the determined ROS photogeneration efficiencies are most probable due to the different electron acceptor capacities of the iron oxide phases present in the nanocomposite samples.

The OH· radicals are known as powerful oxidants, exerting both cytotoxic and pollutant decomposition action, being frequently employed as active agents in antitumor, antimicrobial and water decontamination applications^2,69,70^. In regard to the photocatalytic decomposition of pollutants in wastewaters^70^, the dual magnetic and ROS photogeneration properties of the nanocomposites, as well as their composition consisting of maghemite and TiO_2_, which are both considered nanomaterials with good biocompatibility^11,25–28^, make them promising magnetic photocatalysts which can be conveniently removed from the treated water by magnetic separation.

The adsorption of serum albumin onto the pristine surface of TiO_2_ hydroxylated in aqueous medium was discussed in several previous studies^71–74^. Albumin has its isoelectric point around pH 4.4–5^75^. Thus, at pH > 5 albumin has negative net charge. The adsorption of albumin onto the surface of the nanocomposites during the ultrasonication in supplemented culture medium took place at pH 7.4. Since TiO_2_ acquires a negative surface charge in alkaline environments, including culture media^76–78^, a dominant electrostatic attraction between albumin and the surface of the nanocomposites would seem unlikely. However, albumin was shown to bind to negatively charged surfaces through the positively charged amino acid residues (lysine, histidine) found in its structure^79^. Although under ordinary conditions the outer layer of albumin is hydrophilic^80^, it also contains hydrophobic domains through which it can bind to hydrophobic surfaces^79^. Our results suggest that the adsorption of albumin onto the nanocomposites occurs through the hydrophilic –C=O bonds of the amide I group through hydrogen bonding^58,59^.

The presence of the hydrophobic groups resulting from the Ti precursor used in the synthesis of the nanocomposites hinders thus the adsorption of albumin onto the samples with low Fe/Ti ratio (high TiO_2_ content).

Another factor with diminishing effect on the adsorption of albumin by the samples with low Fe/Ti ratio is the high curvature of the small sized TiO_2_ nanoparticles found in their composition. It has been shown that highly curved nanoparticle surfaces hinder albumin adsorption^81^. Moreover, the high curvature prevents the denaturation of the adsorbed proteins, albumin keeping thus its hydrophilic form, while the lower curvature of larger nanoparticles favors it^82^. Consequently, the sample NC-PP-50/400 with the highest TiO_2_ content showed the lowest rate of albumin adsorption.

The average mesopore diameter of the PP IONPs-based nanocomposites shows a positive correlation with the BSA adsorption efficiency. This behavior can be explained considering that BSA can adsorb onto mesoporous TiO_2_ nanomaterials only if the average pore size is larger than the hydrodynamic radius of albumin^66^. The Stokes radius of BSA was determined under various conditions and the reported values fall between 3 and 9 nm^65,66,83^. Thus, while albumin adsorption is clearly favored by the pore size in the case of NC-PP-500/400, the small pores of the NC-PP-50/400 sample may hinder it. Moreover, NC-PP-50/400 shows the lowest BET surface area and total pore volume (Table 4), these characteristics also diminishing the albumin adsorption. In the case of the NC-PP-200/400 composite, BSA adsorption is favored by the large surface area and total pore volume and hindered by the surface hydrophobic groups. The average pore size of NC-PP-200/400 may be comparable to—or larger than—the BSA Stokes radius.

The fact that albumin is present on the NCs surface corroborates with the results of the in vitro study. The higher the Fe/Ti ratio (implying higher albumin coating), the higher the agglomeration of nanocomposites on the surface of the cultured cells, especially NIH 3T3 (Fig. 17). These observations confirm the enhanced interaction between NIH 3T3 cells and albumin covered surfaces previously reported by Webb et al.^84^. The involvement of albumin in the nanocomposite-cell interaction is also supported by two other arguments. First, serum albumin is known to bind to the surface of different types of cultured normal and neoplastic cells through albumin binding proteins^85^ and second, in the absence of cells the dispersion of the nanocomposites in the culture medium is homogeneous (Fig. 17, top panels).

The enhanced nanocomposite–cell interaction in the case of the NIH 3T3 cells led to a small decrease of their viability (Figs. 15 and 16). The viability decrease was directly correlated with the amount of albumin adsorbed by each composite sample, being statistically different when compared to the control cells in the case of the NC-PP-500/400 core–shell structure. It has been shown that protein corona can increase nanoparticle toxicity when the adsorbed proteins become denatured^57^ and it is also known that TiO_2_ can induce conformational changes in the secondary structure of adsorbed albumin^86^. Moreover, the lower curvature of the core–shell nanocomposites, given by the large size of the iron phase core, favors protein denaturation. It is thus plausible to argue that the slight toxicity of the NC-PP-500/400 sample is due to the denaturation of the proteins adsorbed onto its surface rather than to the physicochemical properties of the TiO_2_ shell. The biocompatibility of the TiO_2_ phase is confirmed by the lack of toxicity on both cell lines of the NC-PP-50/400 sample which contains the largest amount of TiO_2_.

It is known that nanomaterials with low surface area and negative or neutral surface charge are, in general, less toxic than those positively charged and with large surfaces^87^. Thus, the lack of toxicity of the NC-PP-50/400 composite is also supported by its small BET surface and negative zeta potential. Moreover, the positive correlation between the average pore diameters of the nanocomposites and their low cytotoxic effect revealed in the case of the NIH 3T3 cells comes in addition to the above considerations regarding protein adsorption and denaturation.

The HS 27 human fibroblasts kept their viability close to that of the control cells up to 150 µg/ml of NCs, irrespective of the Fe/Ti ratios. The NCs showed to be less toxic to human fibroblast than to the murine ones. This observation is in accordance with the Mannerstrom et al.^88^ report showing the higher sensitivity of BALB 3T3 mouse fibroblasts to toxic molecules than the BJ human fibroblasts.

Considering the overall results of the present study, we can conclude that sample NC-PP-200/400, showing the highest hyperthermic efficiency and ROS photogeneration activity among the studied samples as well as low in vitro toxicity in the absence of the activating factors, has the highest potential towards the proposed applications.

Although the present preliminary results look very promising, the bioactive properties and the biocompatibility of the proposed iron-oxides/TiO_2_ nanocomposites can be further improved or purpose-oriented, by the tuning of the synthesis parameters or by surface functionalization.

## Conclusions

Iron oxide-TiO_2_ nanocomposites with dual properties, AC magnetic heating under applied alternating magnetic field and reactive oxygen species generation under UVA irradiation, were synthesized using an inexpensive and simple method.

The microscopic structure of the obtained materials, ranging from core–shell structures to solid dispersions of iron oxide nanoparticles embedded in TiO_2_ matrices or randomly mixed aggregates of iron oxide and TiO_2_ nanoparticles, is dictated by the particle size and ratio of the iron and titanium phases. The strength of the nanocomposite magnetic and photocatalytic properties depends on both the microscopic phase distribution and the thermal treatment temperature used in the synthesis process. In order to achieve the desired double functionality of the nanocomposites, the thermal treatment temperature should be high enough to ensure the crystallization of the anatase TiO_2_ phase and low enough to avoid the massive formation of the weakly ferromagnetic α-Fe_2_O_3_ phase. In the present study, the appropriate temperature was found to be in the range 400–450 °C. The obtained nanocomposites showed convenient magnetic and photocatalytic properties as well as good in vitro cytocompatibility with regard to human fibroblasts.

The overall results indicate that it is feasible to synthesize multipurpose iron oxide-TiO_2_ magnetic nanocomposites with engineered morpho-structural, AC magnetic heating, ROS photogeneration and biocompatibility properties, convenient for their use in various biomedical and environment-related applications. The produced nanocomposites are appropriate for further functionalization with organic photosensitizers, therapeutic molecules or pollutant-binding compounds.

## Methods

### Materials synthesis

Three types of iron oxide nanoparticles have been used in the present study: the commercial nanomaterial Fe_3_O_4_-637,106-25G (Sigma-Aldrich) (named SIAL) and two samples synthesized in our laboratory (named PP and COPP).

#### Synthesis of iron oxide by precipitation (PP)

Iron oxide nanoparticles were synthesized starting from iron sulfate (FeSO_4_·7H_2_O, Merck) by precipitation in alkaline (NaOH, 1 M) solution. The FeSO_4_ solution (pH 3) was dropwise added to the NaOH solution until the pH reached the value pH 12. The reaction mixture was bubbled with air for four hours, at 80 °C. The obtained precipitate was washed with distilled water (until pH 6.5–7) and dried in air for 5 h at 80 °C.

#### Synthesis of iron oxide by coprecipitation (COPP)

The following reagents were used as received for the synthesis of the COPP sample: iron (III) chloride (FeCl_3_·6H_2_O, 99%, Merck), iron (II) chloride (FeCl_2_·4H_2_O, ≥ 99%, Merck) and sodium hydroxide (NaOH, ≥ 99%, Merck). The NaOH solution (0.17275 mol, 1.15 M) was dropwise added to the aqueous solution containing FeCl_3_ (0.043 mol, 0.2 M) and FeCl_2_ (0.0215 mol, 0.1 M). The co-precipitation reaction was carried out in an Argon atmosphere under continuous stirring at 60 °C. The precipitate was separated by normal filtration, thoroughly washed with deionized water and absolute ethanol (no chlorine ions were detected in the filtrate, AgNO_3_ solution was used), and finally air-dried for 24 h at 80 °C.

#### Synthesis of iron oxide: TiO_2_ nanocomposites

Equal amounts of SIAL, PP and COPP iron oxide samples were vigorously dispersed by vortexing and bath ultrasonication in titanium butoxide (C_16_H_36_O_4_Ti, TBT) at concentrations of 50, 200 and 500 mg/ml. TBT was afterward hydrolyzed by distilled water added in excess to the iron oxide suspensions. The unreacted water was subsequently discarded and the produced solid precipitates were dried at 80 °C and exposed to thermal treatment (TT) at temperatures between 200 and 600 °C, for one hour, in atmospheric air.

The dispersed phase (iron oxide samples) and dispersion medium (titanium butoxide) were also processed separately, following the same protocols as described above for NPs dispersions.

The following sample naming scheme is used for the nanocomposites (NCs): NC-type of iron oxide (SIAL/PP/COPP)-concentration of iron oxide (mg/ml TBT)/TT temperature. For example, sample NC-PP-200/400 is the nanocomposite obtained using the PP iron oxide with a concentration of 200 mg/ml TBT, treated at 400 °C.

### Materials characterization

#### Morpho-structural and textural characterization

X-ray diffraction (XRD) measurements were performed on three types of samples, before and after thermal treatment at 400 °C and 600 °C: iron oxides (SIAL, PP, COPP), TiO_2_ precipitates, and iron oxide-TiO_2_ nanocomposites. Diffraction patterns were recorded with an angular resolution of 0.02° and a scan speed of 0.3 deg min^−1^ on a D8 Advance Powder X-ray Diffractometer from Bruker AXS, at room temperature, using Cu K_α_ radiation (Cu K_α1_ 1.540598 Å and Cu K_α2_ 1.544426 Å). The Rietveld refinement of the XRD data was carried out using the Powder Cell software^89^.

Transmission Electron Microscopy (TEM) images were acquired using the analytical JEOL ARM200F and JEOL 2100 microscopes operated at 200 kV. The microscopes are equipped with a JEOL JED-2300 T unit for Energy-Dispersive X-Ray spectroscopy (EDS) or a Gatan Quantum SE for Electron Energy Loss Spectroscopy (EELS). The samples were observed in conventional TEM (CTEM) at magnifications ranging from 5000 to 300,000. The distribution of the selected chemical elements (Fe, Ti and O) in the sample was analyzed by EDS mapping of EELS in STEM mode. With this method, both spatial and spectral information was acquired simultaneously in each pixel. The signal from each point of the scan was collected by the detector. The powders were gently crushed in an agate mortar and dispersed in ethanol. A droplet of this suspension was then deposited onto a TEM Cu grid.

^57^Fe Mössbauer spectra were recorded at room temperature using a WissEL-ICE Oxford Mössbauer cryomagnetic system and a 10 mCi ^57^Co source in Rh matrix. The velocity calibration of the spectrometer was performed with a high purity α-Fe foil in the velocity range of ± 10 mm/s.

Electron Paramagnetic Resonance (EPR) measurements were carried out with a continuous wave X-Band EPR spectrometer Bruker EMX plus equipped with a Bruker X-SHQ 4119HS-W1 X-band resonator. The measurement parameters for the solid samples, if not otherwise mentioned, were set as follows: microwave frequency 9.87224 GHz, microwave power 15 μW, modulation amplitude 0.1 mT, receiver gain 2 × 10^3^, conversion time 40 ms, time constant 20.96 ms, number of points 1024 and number of scans 1. All EPR measurements were carried out at room temperature.

Fourier Transform Infrared (FTIR) measurements were recorded on a Jasco Spectrometer FT/IR-6600type A equipped with a Standard Light Source and a TGS detector. The spectra were obtained in the 4000–400 cm^−1^ spectral range with 32 scans and a resolution of 4 cm^−1^ using KBr pellets.

In the protein adsorption study, the samples mixed in KBr pellets were prepared as follows: nanocomposites were sterilized (121 °C, 1 h, dry atmosphere), suspended at 2000 µg/ml in either sterile supplemented cell culture medium, Hanks' Balanced Salt Solution (HBSS) (Gibco, 14025) or 0.9% NaCl solution and sonicated on ice bath for 6 h. After sonication, the samples were incubated with supplemented DMEM for 0, 6, 12 and 24 h using the same experimental conditions and procedures used in the cytotoxicity studies described below, but in the absence of cells. Afterwards the NCs were separated by centrifugation (10,000×*g*, 10 min), washed 3 times using HBSS or NaCl solution by ultrasonication and finally dried under vacuum at room temperature for 72 h.

Nitrogen adsorption–desorption isotherms at 77 K were recorded on a Micromeritics ASAP 2020 analyzer (Norcross, GA, USA). The samples were degassed at 200 °C for 4 h under vacuum before analysis. Specific surface areas (S_BET_) were calculated according to the Brunauer–Emmett–Teller (BET) equation, using adsorption data in the relative pressure range between 0.05 and 0.30. The total pore volume (V_total_) was estimated from the amount adsorbed at the relative pressure of 0.99. The pore size distribution curves were obtained from the desorption data using the BJH (Barrett–Joyner–Halenda) model.

Zeta potentials were measured by electrophoretic light scattering (ELS) using a Delsa Nano C particle analyzer (Backman Coulter Brea, CA, USA), with 500 μg/ml sample dispersed by ultrasonication for 30 min in DMEM culture medium supplemented with 10% fetal bovine serum (FBS). The electric field applied on the suspension was fixed at 60 V.

#### Magnetic heating

The efficiency of AC magnetic heating of the synthesized nanocomposites was determined using a RF magnetic inductor (Ambrell EasyHeat, 4.2 KW) operating at 235 kHz, for applied field intensities in the range 80–230 Oe. Homogeneous ferrofluid samples, each with the volume of 1 ml and iron oxide volume fraction φ = 0.02, were obtained by dispersing nanocomposite materials in polyethylene glycol solution (PEG-1000, 25%). Each sample was placed in a polypropylene tube insulated by a PVC vacuum flask, positioned at the center of the helical inductor coil, and exposed to uniform RF fields with different amplitudes (calculated using the COMSOL Multiphysics software). The temperature, T(t), was recorded using an optical fiber thermometer connected to a computer.

#### ROS photogeneration

Spin trapping experiments were carried out using 5,5-Dimethyl-1-pyrroline N-oxide (DMPO) as a spin trapping agent. The samples containing 1 mM TiO_2_ and 10 mM DMPO were irradiated with a UV (365 nm) diode for different time periods. Directly after irradiation, the EPR spectra were recorded with the following parameters: microwave frequency 9.8717 GHz, microwave power 1 mW, modulation amplitude 0.2 mT, receiver gain 2 × 10^3^, conversion time 40 ms, time constant 20.96 ms, number of points 1024 and number of scans 5.

#### In vitro cytocompatibility

##### Cells

Two cell lines of fibroblasts were used: (i) murine embryonic NIH 3T3 (ATCC-CRL 1658) and (ii) human foreskin HS 27 (ATCC CRL 1634), both cultured in high glucose Dulbecco’s Modified Eagle Medium (DMEM, Biochrom F0445, Germany) supplemented with 2 mM l-glutamine (G7513-100 ml, Sigma-Aldrich, UK) and 10% Fetal Bovine Serum (Sigma-Aldrich, F7524, EU). Both cell lines were cultured at 37 °C in a humidified 5% CO_2_ atmosphere.

##### Cytotoxicity assay (MTS)

CellTiter 96 Aqueous One Solution Cell Proliferation Assay kit (Promega G3581, USA) was used to measure the cell viability. Viable cells reduce the reagent 3-(4,5-dimethylthiazol-2-yl)-5-(3-carboxymethoxyphenyl)-2-(4-sulfophenyl)-2H-tetrazolium salt (MTS) to the soluble formazan, whose concentration can be photometrically measured (maximum absorption at 490 nm). The formazan concentration depends linearly on the number of viable cells.

##### Cells exposure to NCs

The nanocomposites (NC-PP-500/400, NC-PP-200/400, NC-PP-50/400) were sterilized (121 °C, 1 h, dry atmosphere), suspended at 2000 µg/ml in either supplemented culture medium or HBSS and sonicated on ice bath for 6 h.

The cells were seeded in 96-well plates (TPP 92196, Switzerland) at 6000 cells/well for HS 27 and 4000 cells/well for NIH 3T3 and grown for 24 h (at 37 °C in humidified 5% CO_2_ atmosphere). The NCs suspensions were added to the cells and final concentrations of: 0, 10, 25, 50, 75, 100, 125, 150 µg/ml were obtained. To account for technical and biological variability, all samples and controls were prepared in four identical wells for each NCs concentration and the tests were performed in triplicate.

After further 24 h of incubation, the medium was removed, 180 µl of a mixture culture medium and MTS (volume ratio 5:1) were added to each well and cells were incubated for 2 h at 37 °C. 150 µl of clear supernatant were afterwards collected from each well and the absorbance was measured at 490 nm using a plate reader (Awareness Technology Inc., Taiwan). The absorbance values of each well was divided by the averaged absorbance value of the controls (0 µg/ml NCs). The results are presented as percentages to the controls with standard deviations.

Tests without cells were also performed to identify any experimental artifacts induced by physicochemical interactions between the NCs and the MTS reagent. No interference of NCs with the MTS reaction was observed (data not shown).

The statistical significance of the data was assessed by means of Kruskal–Wallis test (p < 0.05 was considered statistically significant) using OriginPro™ for FluorEssence™2.1 (Horiba).

## Data Availability

The raw XRD experimental data are available at Science Data Bank Repository as: Ioana Dorina Vlaicu. XRD-Scientific Reports. (2022) 10.11922/sciencedb.01633.
